# TCF-1 regulates HIV-specific CD8^+^ T cell expansion capacity

**DOI:** 10.1172/jci.insight.136648

**Published:** 2021-02-08

**Authors:** Rachel L. Rutishauser, Christian Deo T. Deguit, Joseph Hiatt, Franziska Blaeschke, Theodore L. Roth, Lynn Wang, Kyle A. Raymond, Carly E. Starke, Joseph C. Mudd, Wenxuan Chen, Carolyn Smullin, Rodrigo Matus-Nicodemos, Rebecca Hoh, Melissa Krone, Frederick M. Hecht, Christopher D. Pilcher, Jeffrey N. Martin, Richard A. Koup, Daniel C. Douek, Jason M. Brenchley, Rafick-Pierre Sékaly, Satish K. Pillai, Alexander Marson, Steven G. Deeks, Joseph M. McCune, Peter W. Hunt

**Affiliations:** 1 Department of Medicine, UCSF, San Francisco, California, USA.; 2 Institute of Human Genetics, University of the Philippines-National Institutes of Health, Manila, Philippines.; 3 Department of Microbiology and Immunology,; 4 Medical Scientist Training Program,; 5 Biomedical Sciences Graduate Program, and; 6 Diabetes Center, UCSF, San Francisco, California, USA.; 7 Innovative Genomics Institute, University of California, Berkeley, Berkeley, California, USA.; 8 Vitalant Research Institute, San Francisco, California, USA.; 9 Department of Laboratory Medicine, UCSF, California, USA.; 10 Barrier Immunity Section, Laboratory of Viral Diseases and; 11 Human Immunology Section, Vaccine Research Center, National Institute of Allergy and Infectious Diseases, NIH, Bethesda, Maryland, USA.; 12 Department of Epidemiology and Biostatistics, UCSF, San Francisco, California, USA.; 13 Immunology Laboratory, Vaccine Research Center, National Institute of Allergy and Infectious Diseases , NIH, Bethesda, Maryland, USA.; 14 Department of Pathology, Case Western Reserve University, Cleveland, Ohio, USA.; 15 Chan Zuckerberg Biohub, San Francisco, California, USA.; 16 UCSF Hellen Diller Family Comprehensive Cancer Center, UCSF, San Francisco, California, USA.; 17 Parker Institute for Cancer Immunotherapy, San Francisco, California, USA.

**Keywords:** AIDS/HIV, Immunology, Adaptive immunity, T cells

## Abstract

Although many HIV cure strategies seek to expand HIV-specific CD8^+^ T cells to control the virus, all are likely to fail if cellular exhaustion is not prevented. A loss in stem-like memory properties (i.e., the ability to proliferate and generate secondary effector cells) is a key feature of exhaustion; little is known, however, about how these properties are regulated in human virus–specific CD8^+^ T cells. We found that virus-specific CD8^+^ T cells from humans and nonhuman primates naturally controlling HIV/SIV infection express more of the transcription factor TCF-1 than noncontrollers. HIV-specific CD8^+^ T cell TCF-1 expression correlated with memory marker expression and expansion capacity and declined with antigenic stimulation. CRISPR-Cas9 editing of TCF-1 in human primary T cells demonstrated a direct role in regulating expansion capacity. Collectively, these data suggest that TCF-1 contributes to the regulation of the stem-like memory property of secondary expansion capacity of HIV-specific CD8^+^ T cells, and they provide a rationale for exploring the enhancement of this pathway in T cell–based therapeutic strategies for HIV.

## Graphical abstract

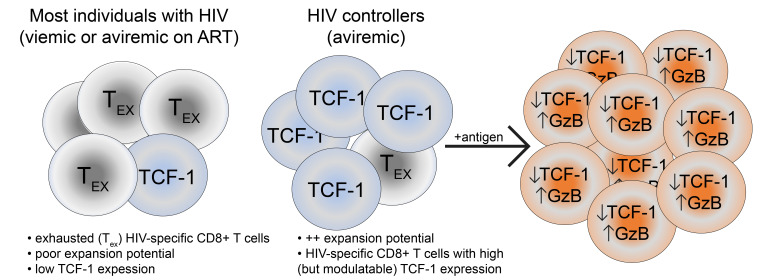

## Introduction

In the vast majority of individuals, infection with human immunodeficiency virus-1 (HIV) leads to chronic viremia that is not controlled by natural host immune responses. The failure of the immune system to control HIV is multifactorial, but one important component is the exhaustion of HIV-specific CD8^+^ T cells (1–3). Antigen-specific CD8^+^ T cell exhaustion occurs in the setting of chronic antigen exposure, and exhausted CD8^+^ T cells are defined by their impaired proliferation, effector cytokine production, and killing capacity after T cell receptor (TCR) stimulation with peptide (4). Therefore, even though they can initially control the viral load (VL) (5–9), HIV-specific CD8^+^ T cells that persist during the chronic phase of infection fail to eradicate infected cells from the body. Generating long-lived, nonexhausted antigen-specific CD8^+^ T cells that have stem-like T cell memory properties (i.e., the capacity to proliferate and to generate a burst of cytotoxic effector cells upon encountering cognate antigen) is the focus of several immunotherapeutic strategies (e.g., therapeutic vaccination, blockade of coinhibitory receptors such as PD-1, and adoptive T cell therapies) for HIV (10–12) and other diseases in which CD8^+^ T cell exhaustion is observed, such as other chronic infections and cancers (4).

Unlike most individuals with HIV who have high VLs in the absence of treatment with antiretroviral therapy (ART), roughly 1% of individuals with HIV, termed “controllers” here (also referred to as “elite controllers”), naturally control infection to undetectable levels in the blood in the absence of ART, despite continuing to harbor the HIV virus in lymphoid tissues throughout the body (13). Several lines of evidence (e.g., genomic associations between HIV control and peptide-binding domain sequence variants in MHC Class I alleles and data from nonhuman primates infected with SIV infection showing that controller animals experience viral rebound after CD8^+^ T cell depletion; refs. 14–18) suggest that an effective HIV-specific CD8^+^ T cell response may contribute to the natural control of HIV in these individuals. Furthermore, compared with HIV-specific CD8^+^ T cells from either viremic or ART-suppressed noncontrollers, HIV-specific CD8^+^ T cells isolated from controllers demonstrate stem-like memory T cell properties upon in vitro stimulation with peptide (19–24). Thus, controllers offer a model for understanding the mechanisms that support the generation of functional stem-like memory CD8^+^ T cell responses in humans in the context of a chronic infection. While several cell-intrinsic factors — including the expression of PD-1 (1, 2) and other coinhibitory receptors (25–27), activation of caspase-8 (28), and the expression of the transcription factor BATF (29) — are known to regulate HIV-specific CD8^+^ T cell dysfunction, little is known about the molecular pathways that support the stem-like memory properties observed in HIV-specific CD8^+^ T cells from controllers.

In recent years, the Wnt-signaling transcription factor T cell factor 1 (TCF-1; also known by its gene name, *TCF7*) (30) has been recognized as an important regulator of antigen-specific CD8^+^ T cell memory stem-like properties of expansion and regenerative capacity (31–33). TCF-1 is highly expressed in naive, central memory (TCM), and stem cell memory CD8^+^ T cells (34). In murine acute infection models, TCF-1 expression is required for the formation of long-lived antigen-specific memory responses that have the capacity to reexpand upon secondary challenge (31–33, 35). In the setting of chronic lymphocytic choriomeningitis (LCMV) infection in mice, TCF-1 marks the subpopulation of stem-like virus–specific CD8^+^ T cells that is capable of regenerating both the ongoing effector cell response and the proliferative burst of effector cells that is observed after blockade of the PD-1 signaling pathway (36–38). In humans, expression of TCF-1 in subpopulations of virus-specific CD8^+^ T cells has been reported in individuals chronically infected with hepatitis B virus (HBV) (39), hepatitis D virus (HDV) (40), or Epstein-Barr virus (EBV) (40), and TCF-1 expression has been correlated with virus-specific T cell expansion capacity in the context of infection with hepatitis C virus (HCV) (37, 41). Furthermore, in several recent clinical trials of individuals with melanoma, the size of the TCF-1–expressing subpopulation of intratumoral CD8^+^ T cells was strongly correlated with the clinical response to checkpoint blockade therapy (42, 43). At a mechanistic level, transcriptional programs supported by TCF-1 may counteract terminal differentiation and exhaustion programs mediated by other transcription factors (36, 44). In the context of HIV infection, TCF-1 expression has been described in bulk CD8^+^ T cells that reside in the follicular regions of lymph nodes (45) and in HIV-specific CD8^+^ T cells (46). To date, the relationship between TCF-1 expression and HIV-specific CD8^+^ T cell functional capacity has not been described, nor has a causal relationship been established between TCF-1 and the stem-like memory property of secondary expansion capacity in human virus–specific T cells. Here, we uncover key molecular differences in HIV-specific CD8^+^ T cells from HIV controllers and noncontrollers by exploring the relationship between TCF-1 expression and HIV control and demonstrating a role for this transcription factor in supporting stem-like memory properties in HIV-specific CD8^+^ T cells in humans.

## Results

### HIV- and SIV-specific CD8^+^ T cells from natural controllers have high TCF-1 expression.

**Figure 1:**
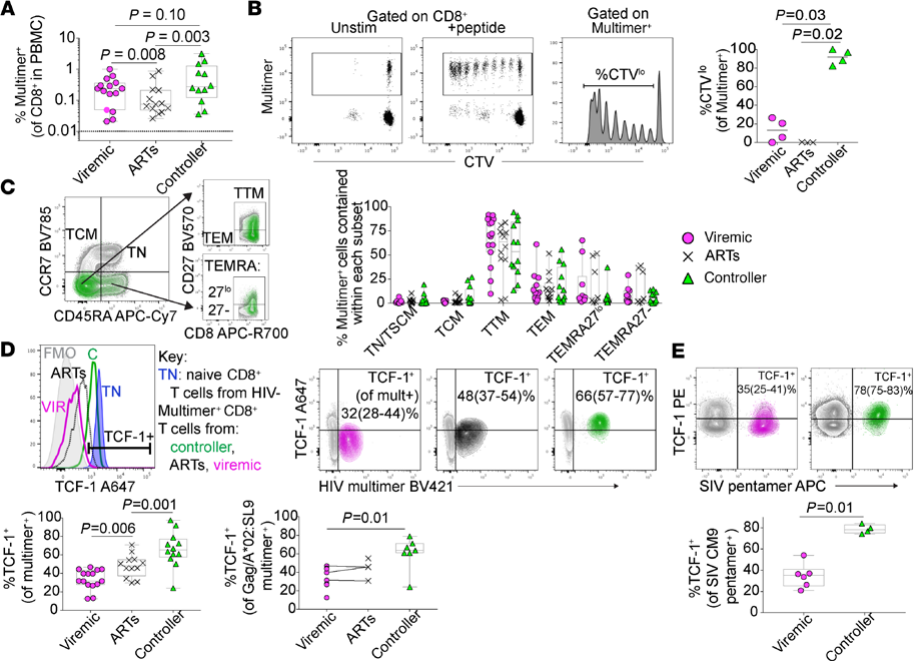
TCF-1 expression is elevated in HIV- and SIV-specific CD8^+^ T cells from controllers. (**A**) Frequency of peripheral blood multimer^+^ HIV–specific CD8^+^ T cells. (**B**) Proliferation of HIV-specific CD8^+^ T cells in response to 6-day in vitro cognate peptide stimulation as measured by dilution of cell-trace violet (CTV). (**C**) Gating strategy (left: green, multimer^+^ from controller; gray, bulk CD8^+^ T cells) and distribution (right) of effector-memory phenotypes amongst multimer^+^ cells (TN, naive [gate includes, and likely primarily contains, CD95^+^ stem-cell memory cells, TSCM]; TCM, central memory; TTM, transitional memory; TEM, effector memory; TEMRA, effector memory-RA, separated by level of CD27 expression). (**D**) Gating (top left; TCF-1^+^ population gated based on CD8^+^ TN population from an HIV-uninfected participant, blue), representative flow plots (top right; median [range]), and summary data (bottom) showing TCF-1 expression in multimer^+^ HIV–specific CD8^+^ T cells from viremic (VIR; magenta), ART-suppressed (ARTs; black), and controller (C; green) individuals of all multimer specificities (left) and within the HIV Gag/HLA-A*02:SL9 multimer^+^ population (right). (**E**) TCF-1 expression in the SIV Gag/Mamu-A*01:CM9 multimer^+^ population from viremic and controller macaques. Phenotypes assessed by flow cytometry. FMO, fluorescence-minus-1 control. Box plots: median ± IQR. The human studies included data from a maximum of *n* = 13 viremic, 10 ART-suppressed, and 12 controller participants (as indicated in each figure), some with 2 multimer specificities. The macaque studies included *n* = 6 viremic and 4 controller animals. Linear mixed effects models to account for clustering within participants (**A**, **C**, **D**), Kruskal-Wallis followed by Dunn’s multiple comparison testing (**B**), Wilcoxon’s rank sum (**E**) were used.

We first sought to confirm in our cohort the observation that HIV-specific CD8^+^ T cells from individuals who naturally control HIV infection to undetectable levels in the blood off of ART (“controllers”) have enhanced proliferative capacity (19, 21, 28). To do this, we identified HLA-typed participants with HIV enrolled in the San Francisco–based SCOPE cohort (https://hividgm.ucsf.edu/scope-study; ClinicalTrials.gov, NCT00187512) who had detectable HIV-specific CD8^+^ T cell responses in the peripheral blood by MHC Class I multimer staining (“multimer^+^”). Participants were sampled from 1 of 3 clinical groups: controller, viremic, or ART-suppressed (*n* = 12, 13, and 10 participants in each group, respectively; see Methods for definitions) (Figure 1A and Supplemental Figure 1; supplemental material available online with this article; https://doi.org/10.1172/jci.insight.136648DS1). We labeled peripheral blood mononuclear cells (PBMCs) from these individuals with the cell proliferation dye CellTrace Violet (CTV) and stimulated them in vitro for 6 days with peptide pools containing the peptide recognized by the multimer^+^ CD8^+^ T cell population. As others have reported, we found that the multimer^+^ HIV–specific CD8^+^ T cells from controllers proliferated more robustly and tended to demonstrate a greater absolute increase in the proportion of Granzyme B–expressing cytotoxic effector cells after stimulation compared with multimer^+^ cells from viremic or ART-suppressed individuals (Figure 1B and Supplemental Figure 2). We also noted that the enhanced proliferative capacity of HIV-specific CD8^+^ T cells from controllers in our cohort did not appear to be explained by an increased frequency of cells with a TCM phenotype (CD45RA^–^CCR7^+^; Figure 1C) (47). Instead, multimer^+^ cells evaluated directly ex vivo across clinical groups tended to fall into a transitional memory phenotype (TTM; CD45RA^+^CCR7^–^CD27^+^), regardless of the presence or mechanism of viral control.

Given the association between high TCF-1 expression and the regenerative capacity of antigen-specific CD8^+^ T cells in mice (32, 35), we hypothesized that this transcription factor would be more highly expressed in HIV-specific CD8^+^ T cells from controllers. Indeed, we found that the proportion of multimer^+^ HIV–specific CD8^+^ T cells expressing TCF-1 was highest in controllers, followed by ART-suppressed, and then viremic participants (median 62% versus 51% versus 35% TCF-1^+^, respectively; *P* = 0.006 viremic versus ART-suppressed, *P* = 0.001 ART-suppressed versus controller; Figure 1D). This observation held true, even when we restricted our analysis to a single multimer response (HIV-specific CD8^+^ T cells that recognize the Gag-derived peptide SLYNTVATL presented in the context of HLA-A*02). Compared with viremic individuals, controllers also had a higher frequency of TCF-1^+^ cells within the predominant TTM and TEM subsets of multimer^+^ cells (Supplemental Figure 3), confirming that the higher expression of TCF-1 in multimer^+^ cells from controllers is not simply explained by differential memory subset distribution.

Finally, rhesus macaques (RM) that control SIV in the absence of therapy also have highly functional, relatively nonexhausted SIV-specific CD8^+^ T cells (48). We found that ART-naive controller macaques with low VLs (<1,000 copies/mL) had higher expression of TCF-1 in their Mamu-A*01–restricted CM9 SIV-specific CD8^+^ T cells compared with untreated animals with high VLs (median 290,500 copies/mL; *P* = 0.01; Figure 1E and Supplemental Table 2). Therefore, across 2 primate species, natural control of chronic retroviral infection is associated with the generation of a virus-specific CD8^+^ T cell population with high TCF-1 expression.

### TCF-1–expressing HIV-specific CD8^+^ T cells are phenotypically less differentiated.

**Figure 2:**
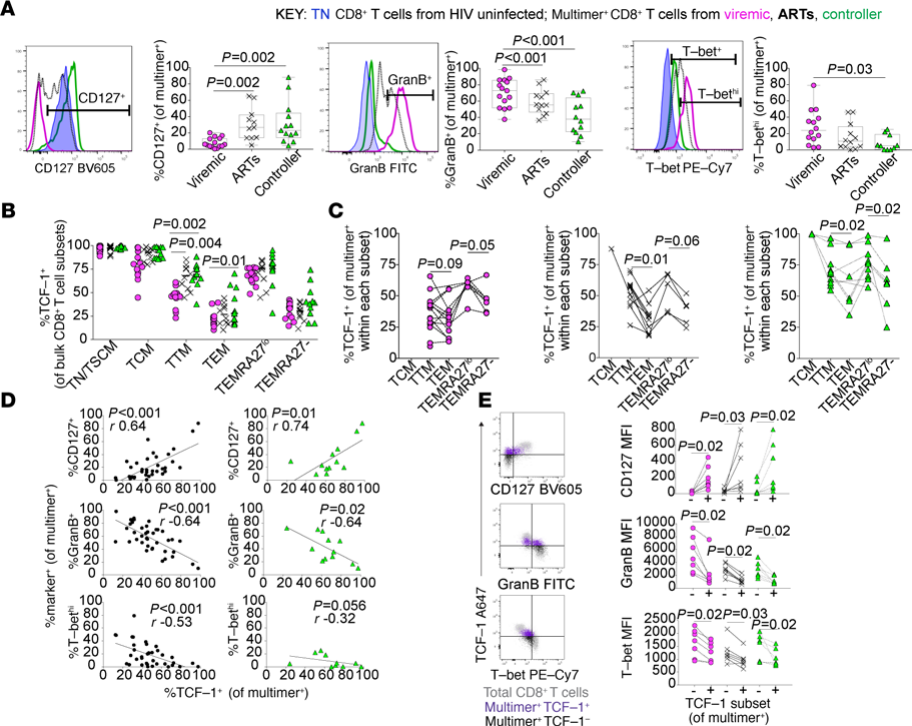
High TCF-1 expression in HIV-specific CD8^+^ T cells is associated with a memory-like phenotype. (**A**) Phenotype of HIV-specific multimer^+^ CD8^+^ T cells from viremic, ART-suppressed, or controller individuals (compared with naive CD8^+^ T cells from an HIV negative individual). (**B** and **C**) TCF-1 expression is higher in less differentiated naive and effector-memory subsets of bulk (**B**) and HIV-specific (**C**) CD8^+^ T cells. (**D**) Correlation between the expression of TCF-1 and other phenotypic markers in multimer^+^ CD8^+^ T cells (GranB, Granzyme B; black dots, all participants; green triangles, controllers only). (**E**) Expression of CD127, Granzyme B, and T-bet within TCF-1^+^ versus TCF-1^–^ HIV-specific CD8^+^ T cells. These studies included data from a maximum of *n* = 13 viremic, 10 ART-suppressed, and 12 controller participants (as indicated in each figure), some with 2 multimer specificities. Linear mixed effects models to account for clustering within participants (**A** and **B**), Wilcoxon’s signed-rank test (**C** and **E**), Spearman’s correlation (**D**).

Although we found that the distribution of classic effector-memory phenotypes within the HIV-specific CD8^+^ T cell population is not affected by the presence or mechanism of viral control in our cohort (Figure 1C), multimer^+^ CD8^+^ T cells evaluated directly ex vivo from HIV controllers had several other phenotypic features more consistent with resting TCM; a larger proportion expressed CD127 while a smaller proportion expressed the effector protein Granzyme B or high levels of the effector differentiation transcription factor T-bet (Figure 2A). In contrast, viremic participants tended to have a more effector/effector-memory–like phenotype of multimer^+^ CD8^+^ T cells. Eomesodermin, another T-box transcription factor important for memory differentiation (49, 50), did not appear to be differently expressed in HIV-specific CD8^+^ T cells from controllers (Supplemental Figure 4A). Similar to humans, SIV-specific CD8^+^ T cells from controller compared with typical progressor macaques with high VLs had a more resting memory–like phenotype (i.e., they had a higher expression of CD127 and LEF-1, a transcription factor that can coordinate with TCF-1 to activate Wnt signaling, and a trend toward lower expression of Granzyme B; refs. 30, 35, 51), and they appeared to be less activated (with lower CD69 expression and a trend toward a smaller proportion of Ki-67^+^ cells; Supplemental Figure 4B). Furthermore, the expression of the chemokine receptor CXCR5 was higher in TCF-1^+^ compared with TCF-1^–^ SIV-specific CD8^+^ T cells among viremic animals (*P* = 0.03). This relationship was also observed in the 4 controller animals, with a trend (*P* = 0.125) toward significance (Supplemental Figure 4B).

In adults without HIV infection, TCF-1 expression has been found to be higher in less-differentiated bulk CD8^+^ T cell subsets (i.e., TN and TCM; ref. 52). We observed a similar pattern in our cohort of participants with HIV in both bulk (Figure 2B) and HIV-specific CD8^+^ T cells (Figure 2C). In particular, CD8^+^ T cell subsets that express CD27, a tumor necrosis factor family protein important for T cell survival (53, 54) (e.g., TN, TCM, and TTM cells, as well as cells in the more terminally differentiated/CD45RA-expressing TEMRA subset that express low levels of CD27), tended to express more TCF-1 compared with subsets that do not express CD27 (e.g., TEM and CD27^–^ TEMRA cells). Furthermore, mirroring the antigen-specific population, controllers also had higher TCF-1 expression within the gated TTM and TEM bulk CD8^+^ T cell subsets compared with viremic individuals (Figure 2B).

Within the population of multimer^+^ HIV–specific CD8^+^ T cells, we found that TCF-1 expression was associated with patterns of phenotypic marker expression typically found in CD8^+^ TCM, with a positive correlation between TCF-1 and CD127 expression (Spearman’s correlation, *r* = 0.64, *P* < 0.0001) and a negative correlation between TCF-1 and Granzyme B (*r* = –0.64, *P* < 0.001), and T-bet expression (*r* = –0.53, *P* < 0.001), both across the whole cohort and specifically within HIV-specific CD8^+^ T cells isolated from controllers (Figure 2D). Finally, TCF-1–expressing HIV-specific CD8^+^ T cells in all 3 groups expressed significantly more CD127 and less Granzyme B and T-bet (Figure 2E), as well as more of the memory-associated transcription factor FOXO1 (per-cell total protein level; Supplemental Figure 4C).

### Varied coinhibitory receptor expression on HIV-specific CD8^+^ T cells from controllers.

**Figure 3:**
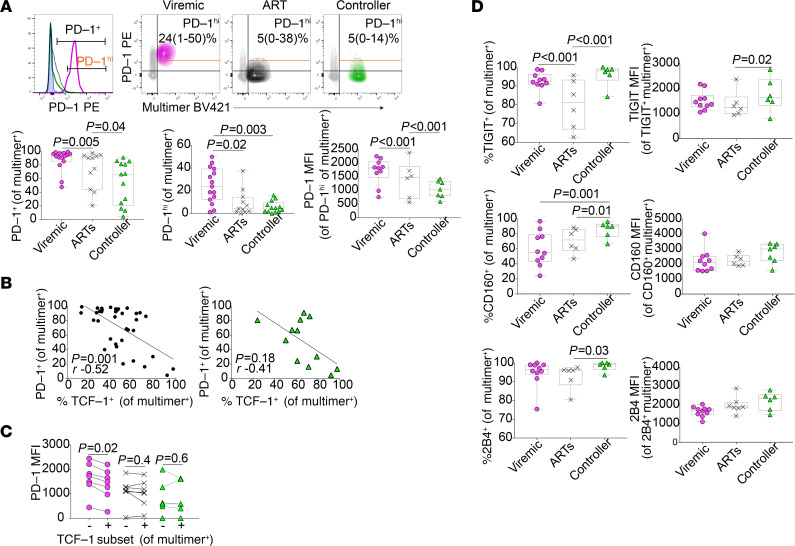
HIV-specific CD8^+^ T cells have lower expression of PD-1 but not other coinhibitory receptors. (**A**) Expression of PD-1 in multimer^+^ HIV–specific multimer^+^ CD8^+^ T cells (annotated with median and range; MFI, median fluorescence intensity). (**B**) Correlation between PD-1 and TCF-1 expression in multimer^+^ HIV-specific CD8^+^ T cells (black dots, all participants; green triangles, controllers only). (**C**) Expression of PD-1 within TCF-1^+^ versus TCF-1^–^ HIV-specific CD8^+^ T cells. (**D**) Expression of TIGIT, CD160, and 2B4 in multimer^+^ HIV–specific multimer^+^ CD8^+^ T cells. These studies included data from a maximum of *n* = 13 viremic, 10 ART-suppressed, and 12 controller participants (as indicated in each figure), some with 2 multimer specificities. Linear mixed effects models to account for clustering within participants (**A**, **D**), Wilcoxon’s signed-rank (**C**), and Spearman’s correlation (**B**).

A number of cell surface coinhibitory receptors (e.g., PD-1, TIGIT, CD160, and 2B4) are upregulated in the setting of chronic TCR stimulation, and their expression has been associated with functional exhaustion of antigen-specific CD8^+^ T cells in HIV disease (1, 2, 25, 55–57) and other settings (4). Consistent with their observed robust capacity to proliferate and generate secondary effector cells in response to stimulation, HIV-specific CD8^+^ T cells from controllers expressed PD-1 less frequently than those from both viremic and ART-suppressed individuals (Figure 3A). Although some controllers had a large subpopulation of multimer^+^ CD8^+^ T cells that expressed some PD-1, the absolute level of expression of PD-1 on multimer^+^ cells from controllers was low (as measured by the median fluorescence intensity [MFI], as well as the proportion of HIV-specific CD8^+^ T cells that express high levels of PD-1, [PD-1^hi^]; Figure 3A). Across the cohort, the proportion of HIV-specific CD8^+^ T cells that expressed TCF-1 negatively correlated with the proportion expressing PD-1, and the directionality (but not significance) of this relationship was also maintained when evaluating controllers alone (Figure 3B). TCF-1–expressing cells from viremic individuals have lower expression of PD-1 compared with TCF-1^–^ cells; this relationship, however, did not appear to hold among HIV-specific CD8^+^ T cells from ART-suppressed or controller individuals (Figure 3C). Interestingly, unlike true memory cells generated in the context of acute infection, HIV-specific CD8^+^ T cells from controllers compared with ART-suppressed or viremic individuals did not express lower levels of 3 other coreceptors that have been associated with antigen-specific CD8^+^ T cell functional exhaustion (TIGIT, CD160, and 2B4; Figure 3D).

### TCF-1 expression in HIV-specific CD8^+^ T cells is downregulated by TCR stimulation.

**Figure 4:**
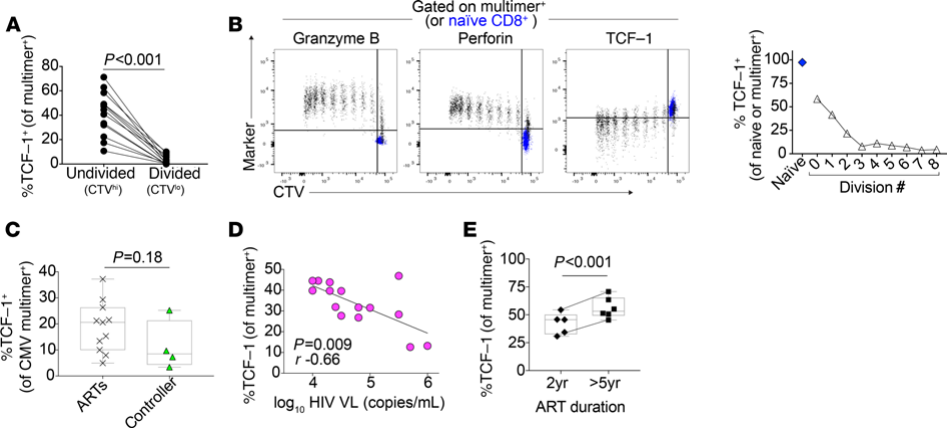
TCF-1 expression is negatively correlated with antigen exposure. (**A**) TCF-1 expression in HIV-specific CD8^+^ T cells after 6 days of in vitro peptide stimulation in divided versus undivided cells (*n* = 12 biological replicates). (**B**) TCF-1 expression in multimer^+^ CD8^+^ T cells (black) in each division peak after 6 days of in vitro peptide stimulation (naive CD8^+^ T cells shown in blue for reference); data are representative of pattern observed in *n* = 12 biological replicates. (**C**) TCF-1 expression in multimer^+^ CMV-specific CD8^+^ T cells from individuals with HIV (ART-suppressed [*n* = 12] versus controller [*n* = 4]). (**D**) Negative correlation between TCF-1 expression and plasma HIV viral load (VL; *n* = 15). (**E**) Expression of TCF-1 in HIV-specific CD8^+^ T cells in ART-suppressed individuals, depending on the duration of ART (*n* = 9 individuals). Wilcoxon’s signed-rank (**A**), Wilcoxon’s rank sum (**C**), Spearman’s correlation (**D**), and linear mixed effects models to account for clustering within participants (**E**).

Because low TCF-1 expression is associated with CD8^+^ T cells that are more differentiated toward an effector fate, we asked whether TCF-1 expression is reduced upon stimulation of HIV-specific CD8^+^ T cells with their cognate peptide to induce secondary effector differentiation. After 6-day in vitro peptide stimulation, the proportion of HIV-specific CD8^+^ T cells expressing TCF-1 decreased in the undivided (CTV^hi^) versus the divided (CTV^lo^) population from a median 44% to 1% (*P* < 0.001; Figure 4A). In a sample where clear division peaks were discernible, this reduction appeared to mostly take place over the course of the first 3 divisions and occurred inversely to the acquisition of secondary effector characteristics (e.g., the expression of Granzyme B and Perforin; Figure 4B).

We next explored how TCF-1 expression in antigen-specific CD8^+^ T cells relates to the level of stimulation they receive from ongoing chronic infection in vivo. We first asked whether the level of TCF-1 in antigen-specific CD8^+^ T cells that recognize a different virus (cytomegalovirus, CMV) is affected by the non–antigen-specific physiologic differences present between the HIV clinical groups (e.g., differences in the levels of cytokines and other inflammatory mediators) (58). We observed a similar level of TCF-1 expression between CMV-specific CD8^+^ T cells observed from ART-suppressed versus controller individuals in a small cohort (Figure 4C). We then asked how different levels of antigen stimulation in vivo relate to TCF-1 expression in HIV-specific CD8^+^ T cells. Among viremic participants, higher TCF-1–expression multimer^+^ HIV–specific CD8^+^ T cells correlated with lower HIV VLs (*r* = –0.66, *P* = 0.009; Figure 4D). The directionality (but not the significance) of this relationship remained in the subset of participants (*n* = 9) with Gag/HLA-A*02:SL9 responses (*r* = –0.40, *P* = 0.28; Supplemental Figure 5), despite the fact that the dominant plasma viral species from all 7 of the participants for whom we have plasma viral sequence data available demonstrated evidence of viral evolution and CD8^+^ T cell epitope escape at this peptide sequence at the time of sampling (Supplemental Table 4).

In contrast to the negative correlation between HIV-specific CD8^+^ T cell TCF-1 expression and VL in viremic participants, we did not observe a significant relationship between HIV cell–associated DNA levels in PBMCs and TCF-1 expression in either ART-suppressed individuals or elite controllers (Supplemental Figure 6). Interestingly, a longer duration of ART (>5 years) was associated with a higher level of TCF-1 expression in HIV-specific CD8^+^ T cells (Figure 4E; *P* < 0.001). Of the controllers we included in our study, we noted that 1 individual had a particularly low frequency of TCF-1–expressing multimer^+^ cells (24%) compared with the rest of the controller group (median 67%; Figure 1D and Supplemental Figure 7). Interestingly, this individual had multiple detectable VL measurements of 100–300 copies/mL starting 3 months after the PBMC sample date (Supplemental Figure 7, right side, bottom). In contrast, the 2 controller individuals with the largest proportion of TCF-1–expressing multimer^+^ cells in the cohort (Supplemental Figure 7, right side, top and middle) have continued to maintain HIV suppression with undetectable VLs in the absence of ART and with frequent testing for 16 and 20 years in the cohort, with the exception of a single detectable VL (<100 copies/mL) more than 8 years prior to sampling in 1 individual.

### TCF-1 regulates human CD8^+^ T cell expansion capacity.

**Figure 5:**
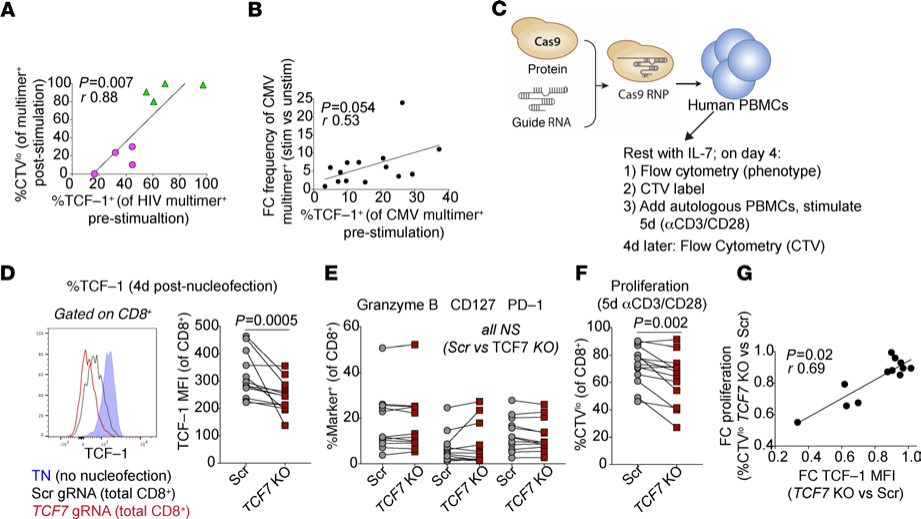
TCF-1 KO impairs human CD8^+^ T cell proliferation. (**A**) Correlation between TCF-1 expression in HIV-specific CD8^+^ T cells and the proportion that divide after 6-day in vitro peptide stimulation. (**B**) Correlation between TCF-1 expression in CMV-specific CD8^+^ T cells and their expansion after 6-day in vitro peptide stimulation (fold change [FC] in the frequency of CMV-specific CD8^+^ T cells [gated on total CD8^+^ T cells], stimulated versus unstimulated cells; *n* = 14). (**C**) CRISPR-Cas9–mediated deletion of *TCF7* (Scr, scrambled guide RNA [gRNA]; RNP, ribonucleoprotein; *n* = 13 biological replicates). (**D**) TCF-1 protein downregulation in CD8^+^ T cells after *TCF7* KO (red) compared with electroporation with Scr gRNA (gray). (**E**) CD8^+^ T cell phenotype after *TCF7* KO. (**F**) Proportion of divided CD8^+^ T cells (%CTV^lo^) after 5-day stimulation with αCD3/CD28. (**G**) Correlation between the reduction in TCF-1 expression (MFI) and the reduction of CD8^+^ T cell proliferation (%CTV^lo^ after stimulation). Spearman’s correlation (**A**, **B**, and **G**) and Wilcoxon’s signed-rank (**D–F**).

We found that TCF-1 expression is associated with a T cell memory–like phenotype in HIV-specific CD8^+^ T cells. We next asked whether TCF-1 expression is correlated with the stem-like memory property of expansion capacity in HIV-specific CD8^+^ T cells. Indeed, we found that the level of TCF-1 expression in multimer^+^ HIV–specific CD8^+^ T cells directly ex vivo correlated with their ability to proliferate in response to 6-day in vitro stimulation with cognate peptide (*r* = 0.88, *P* = 0.007; Figure 5A and Supplemental Figure 8). We also found that multimer^+^ CMV–specific CD8^+^ T cells with higher TCF-1 expression demonstrate a trend toward increased expansion after cognate peptide stimulation (*r* = 0.53, *P* = 0.054; Figure 5B). Interestingly, although a smaller proportion of CMV-specific CD8^+^ T cells expressed TCF-1 compared with HIV-specific CD8^+^ T cells isolated from controllers, their expansion capacity was greater (median 5.4- versus 1.6-fold change increase in frequency, *P* = 0.008).

In order to assess whether TCF-1 directly regulates human CD8^+^ T cell expansion capacity, we employed CRISPR-Cas9–targeted genomic editing approaches to knock out or overexpress the *TCF7* gene in primary human T cells. We first asked whether genetic ablation of *TCF7* impairs proliferation. In comparison with cells electroporated with Cas9 ribonucleoproteins (RNPs) with a control (scrambled) guide RNA not predicted to cut any site in the human genome, CD8^+^ T cells in which *TCF7* was targeted by Cas9 RNPs showed no change in several phenotypic markers but had impaired proliferation and/or survival after 5-day polyclonal TCR stimulation with αCD3/CD28 antibodies (*P* = 0.002; Figure 5, C–F; T cell viability after electroporation shown in Supplemental Figure 9). Indeed, the efficacy of the *TCF7* deletion (as measured by TCF-1 MFI) correlated with the impact of the deletion on proliferative responses (Figure 5G).

**Figure 6:**
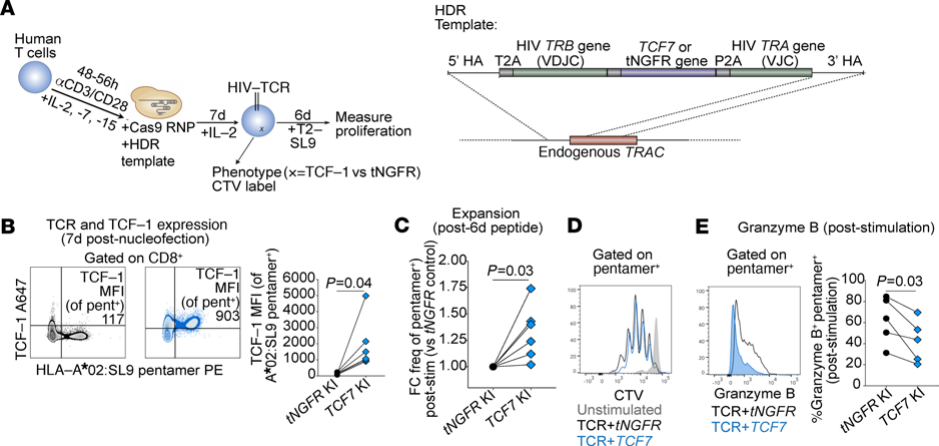
TCF-1 overexpression enhances HIV-specific CD8^+^ T cell expansion after peptide stimulation. (**A**) Generation of HIV-specific T cell receptor (TCR) T cells using CRISPR-Cas9 knock-in (KI) of HIV-specific TCR and *TCF7* (versus truncated Nerve Growth Factor Receptor [*tNGFR*]); T2, HLA-A*02-expressing T2 cell line; SL9, SLYNTVATL peptide. (**B**) TCF-1 protein expression after *tNGFR* (black) or *TCF7* (blue) KI (left, representative flow plots; right, summary TCF-1 MFI from *n* = 6 biological replicates; lines connect control and TCF-1-expressing samples generated from the same donor). (**C**) Frequency of *TCF7* KI TCR T cells after 6-day in vitro stimulation with SL9 peptide loaded on T2 cells (FC versus *tNGFR* KI; *n* = 6 biological replicates). (**D**) CTV tracings of TCR T cells (unstimulated or stimulated). (**E**) Granzyme B expression in *TCF7* KI TCR T cells. Wilcoxon’s signed-rank (**B**, **C**, and **E**).

Using a recently developed nonviral gene knock-in approach, we genetically modified primary human T cells to replace the endogenous TCR with an HIV-specific TCR coexpressed with TCF-1 or an irrelevant control protein, truncated nerve growth factor receptor (tNGFR), both under the control of the endogenous TCRα gene (*TRAC*) promoter (59, 60) (Figure 6, A and B). Six-day in vitro stimulation of these cells with cognate peptide-loaded antigen presenting cells led to an increase in the recovery of expanded TCR T cells that overexpress the TCF-1 protein compared with TCR T cells expressing tNGFR (*P* = 0.03; Figure 6C). Based on CTV tracings, the increased recovery of the *TCF7*–knock-in TCR T cells appeared to be due to enhanced survival of the cells in the culture rather than an absolute increase in the proportion of cells that proliferated (Figure 6D and Supplemental Figure 10). Consistent with its role as a regulator of stem-like memory properties, overexpression of TCF-1 was also associated with a small but significant decrease in the expression of the cytolytic protein, Granzyme B, in HIV-specific TCR T cells (Figure 6E).

## Discussion

The goal of many immune-based strategies that aim to enhance the control of HIV is to elicit functional, nonexhausted, and durable HIV-specific CD8^+^ T cells that have the stem-like memory T cell capacity to rapidly expand into secondary effector cells that can kill HIV-infected cells (10, 12). We hypothesized that TCF-1, a Wnt signaling transcription factor known to regulate T cell “stemness” in several other contexts, might also contribute to the regulation of expansion capacity of HIV-specific CD8^+^ T cells. We found that MHC Class I multimer^+^ HIV–specific CD8^+^ T cells with high proliferative capacity from HIV controllers compared with cells from noncontrollers on or off of ART have a larger TCF-1^+^ subpopulation, that TCF-1 expression in these cells is associated with higher expression of other proteins associated with T cell memory function and lower PD-1 expression, that KO of the *TCF7* gene impairs polyclonal CD8^+^ T cell proliferation, and that *TCF7* gene overexpression in genetically engineered HIV-specific CD8^+^ T cells enhances their accumulation after peptide stimulation. Identifying the pathways that support antigen-specific CD8^+^ T cell stem-like capacity in the context of HIV is critical not only to directly inform the development of improved CD8^+^ T cell–based therapies to control HIV, but also to more broadly understand how this unique differentiation state is regulated in human CD8^+^ T cells.

These data are the first indication, to our knowledge, of a direct role for *TCF7*/TCF-1 in the regulation of expansion capacity in human virus-specific T cells. Our phenotypic findings align with several studies that have demonstrated a role for TCF-1 in supporting memory cell capacity and countering terminal effector differentiation and exhaustion programs in mice with acute and chronic viral infections, in human chronic hepatitis C infection, and in mouse and human cancers (31, 32, 36–38, 41, 44, 61–65). Taken together with our functional studies, these data provide a clear rationale for further exploration of this pathway as a potential target for modulation in T cell–based therapies for HIV. For example, therapeutic vaccine regimens (10) might be improved by employing adjuvants that promote TCF-1 expression in vaccine-elicited HIV-specific CD8^+^ T cells. Similarly, overexpressing TCF-1 in HIV-specific receptor–engineered T cells prepared for adoptive transfer (either TCR T cells, as we have made here, or chimeric antigen receptor [CAR] T cells; ref. 66) might be one approach to overcoming the major challenge of generating T cells that can persist long-term and avoid exhaustion after in vivo infusion (67).

Identifying a mechanistic pathway that supports the stem-like memory T cell functional capacity of HIV-specific CD8^+^ T cells in individuals who naturally control this chronic infection raises 2 important questions. First, is this capacity a cause or a consequence of viral control in these individuals? Because TCF-1 expression decreases in antigen-specific CD8^+^ T cells upon TCR stimulation, it is possible that the higher levels of TCF-1 that we observe in controllers is at least in part a consequence of low levels of antigen exposure in these individuals. Interestingly, we observed low TCF-1 expression in HIV-specific CD8^+^ T cells from viremic noncontrollers even after either CD8^+^ T cell epitope escape or viral suppression with ART. These results suggest that there may be a threshold of high antigen exposure, beyond which — even if there is subsequently a reduction in TCR stimulation — antigen-specific CD8^+^ T cells are unable to recover stem-like memory properties. At the same time, while it is difficult to mechanistically show this in humans, it is also very plausible that the enhanced ability of HIV-specific CD8^+^ T cells from controllers to mount a robust proliferative response (a capacity that is directly linked to their in vitro cytotoxicity) is causally linked to their ability to naturally contain the virus.

A second question raised by our data is whether promoting stem-like memory T cell capacity is the optimal differentiation state to target for T cell–based interventions to treat HIV, a setting in which the therapy-derived T cells must outpace viral rebound after ART discontinuation presumably by (a) being present in high enough numbers, (b) being localized in the correct tissues, and (c) having sufficient (and appropriate) effector functions to eliminate infected cells. TCF-1 is a promising target to improve T cell–based HIV therapies because animal models suggest that TCF-1–regulated stem-like properties are essential for protective CD8^+^ T cell secondary recall responses after natural infection or vaccination (31, 35) and the survival of CD8^+^ T cells that experience chronic stimulation (37, 38). However, our data in TCR T cells genetically engineered to overexpress *TCF7* also support findings in other studies showing that constitutive TCF-1 expression or activation of the Wnt signaling pathway promotes T cell stemness at the expense of effector differentiation (33, 68). In other words, permanent overexpression of TCF-1 might support the expansion capacity of HIV-specific CD8^+^ T cells such that they would be able to expand robustly and survive better upon secondary antigen encounter; however, without the ability to downregulate TCF-1, the daughter cells would potentially lack sufficient cytotoxic effector functions. Even if a therapeutic intervention were designed to generate TCF-1^+^ T cells that had the ability to downregulate TCF-1 and differentiate into effector cells upon antigenic stimulation, because TCF-1^+^ T cells themselves are not effector differentiated or poised for immediate killing, it is possible that their delayed responsiveness would be outpaced by viral rebound.

In addition to these questions, for the development of therapeutic vaccination and adoptive T cell therapies for HIV, it will be essential to understand how targeting the TCF-1 pathway in human T cells affects the formation of tissue resident memory (TRM) and follicular cytotoxic (Tfc) CD8^+^ T cells. It has been proposed that CD8^+^ T cells that occupy these differentiation states would provide a rapid response to nearby reactivated virus because they are localized closer to the sites of HIV reservoir persistence within lymphoid tissues (and, in particular, B cell follicles; 69–71). Indeed, studies have suggested that lymphoid tissue from humans — and nonhuman primates that naturally control retroviral infection — is enriched for CD8^+^ T cells that express the TRM-associated protein CD69 (72), as well as CD8^+^ T cells that express the Tfc-associated chemokine receptor CXCR5 (73–75). Studies in mice suggest that TCF-1 may directly promote the generation of Tfc (38) but inhibit the formation of TRMs (76). Although little is known about the relationship between TCF-1 and the formation of these subsets in humans, in our study, we did observe a correlation between the expression of TCF-1 and CXCR5 in SIV-specific CD8^+^ T cells isolated from the spleens of infected RM. To effectively harness this pathway for T cell therapies for HIV and other diseases such as cancer, it will be important to understand the direct impact of TCF-1 expression on TRM/Tfc differentiation, as well as other T cell migratory properties.

As a mostly cross-sectional study performed on peripheral blood samples in humans and splenocytes from nonhuman primates, our study has some limitations. First, because we have not sampled other tissues, we cannot extrapolate our conclusions to infer how TCF-1 interacts with other cellular phenotypes or functions in tissues. Based on work by other groups, we anticipate that HIV-specific CD8^+^ T cells isolated from lymph nodes (72, 77) and the gut (78, 79) will have substantially different phenotypes compared with those in peripheral blood, and it would be important to assess TCF-1 expression in these tissues in follow-up studies. Second, due to inherent limitations in the CRISPR editing protocols, our functional experiments did not allow us to directly test the role of TCF-1 in regulating stem-like memory capacity in endogenous HIV-specific CD8^+^ T cells. However, as protocols in this field are rapidly developing, we hope we will be able to address this question in the future. Finally, as our comparison between HIV- and CMV-specific CD8^+^ T cells demonstrates, although expansion capacity correlates with TCF-1 expression within antigen-specific CD8^+^ T cell populations from the same viral infection, the activity of other CD8^+^ T cell–intrinsic regulatory pathways likely influences overall expansion capacity in different settings.

Our study provides important rationale to support further investigations into the fundamental biology of TCF-1 activity in human T cells and how to harness this pathway to optimize T cell–based therapies for HIV. Although downstream transcriptional and epigenetic targets of TCF-1 have been studied in developing thymocytes and other immune cell types in mice (80), the pathways via which TCF-1 exerts its effects on virus-specific T cells (in mice or humans) are currently unknown. In therapeutic settings, approaches to overexpress TCF-1 in a manner such that levels can be modulated to allow for T cell differentiation while preserving the positive influence of TCF-1 on T cell longevity should be explored. Finally, it will be critical to study whether high levels of TCF-1 in vaccine-elicited T cells correlate with the long-lasting durability of the response, affect the localization of the antigen-specific T cells, and ultimately promote the ability of a therapeutic vaccine regimen to control viral rebound after antiretroviral treatment discontinuation.

## Methods

### Study design.

Our studies of human samples from individuals infected with HIV were designed as an analysis of retrospectively collected PBMCs from individuals enrolled in an existing cohort. We selected samples for inclusion in the study based on screening cohort participants for MHC Class I multimer^+^ HIV–specific CD8^+^ T cell responses and did not perform a power analysis prior to selecting samples for inclusion. Flow cytometry samples were excluded if viability was < 80%. No blinding was performed. The number of participants included and the number of experiments performed for each assay are indicated below.

### Human study participants and samples.

This study sampled PBMCs retrospectively collected from participants with HIV enrolled in the Zuckerberg San Francisco General Hospital clinic-based SCOPE cohort (and individuals coenrolled in the related Options cohort) who had a documented positive HIV antibody test, who were not on ART at the time of enrollment, and who had HIV-specific CD8^+^ T cell responses detectable by MHC Class I multimer staining (Supplemental Table 1). Individuals were classified into 1 of 3 groups: (a) viremic (plasma VL > 9000 copies/mL; infected for at least 6 months [and greater than 2 years for all but 1 participant] prior to sampling; most individuals were ART naive, but if not, they were off ART for at least 2 years); (b) ART-suppressed (VL < 40 copies/mL; infected and untreated for at least 2 years, and then on ART with suppressed VL for at least 2 years prior to sampling); and (c) controller (VL < 100 copies/mL; infected for at least 2 years, with no VLs > 200 copies/mL for at least 3 months prior to and after sampling; most individuals were ART naive, but if not, they were off ART for at least 2 years). CD4^+^ T cell counts and HIV-1 plasma RNA levels were measured in all cohort participants by CLIA-certified clinical laboratory assays at the time of entry into the cohort and approximately every 3–4 months afterward. This study also sampled deidentified PBMCs from blood donors known to have negative testing for HIV, HCV, and HBV (Stanford Blood Center [https://stanfordbloodcenter.org/research-labs/research-products-and-services/] and Vitalant [https://www.vitalant.org/]). PBMCs were collected and cryopreserved as described previously (81, 82).

### Rhesus macaque characteristics, SIV infection, and rhesus splenocyte flow cytometry.

Splenic mononuclear cell suspensions were obtained from 10 Mamu-A*01^+^ RM (*Macaca mulatta*) infected with either SIVmac239, SIVsmE543, or SIVsmE660 and with established SIV infection or AIDS. Macaques were classified as viremic (>1000 copies/mL; *n* = 6) or controller (<1000 copies/mL; *n* = 4) based on plasma RNA VLs (Supplemental Table 2). Cryopreserved samples were homogenized as described previously (83). Tissue homogenates were washed twice in RPMI 1640 medium supplemented with 10% FBS, 2 mM L-glutamine, and 1% penicillin/streptomycin (HyClone via GE Healthcare Life Sciences). SIV-specific CD8^+^ T cells were identified using Mamu-A*01-CTPYDINQM Gag_181–189_ (CM9) Pro5 MHC Class I Pentamers (ProImmune). Cells were stained with the viability marker LIVE/DEAD Aqua (Thermo Fisher Scientific), and surface antigens were subsequently assessed with the fluorochrome-conjugated antibodies listed in Supplemental Table 3. For detection of intracellular proteins, surface antigen–labeled cells were fixed and permeabilized with the eBioscience Foxp3/Transcription Factor Staining Buffer Set (Thermo Fisher Scientific) and incubated with the fluorochrome-labeled antibodies listed in Supplemental Table 3. All macaque samples were evaluated in a single experiment.

### Human PBMC phenotyping by flow cytometry.

Cryopreserved PBMCs were thawed as described previously (81, 82, 84), and 2 million to 4 million cells were stained with MHC Class I multimers, followed by staining for surface and then intracellular proteins. HIV-specific CD8^+^ T cells were identified via staining with peptide–MHC Class I multimers, either monomers that were provided by RPS (Case Western University, Cleveland, Ohio, USA) and tetramerized as described (85) using BV421 or PE, or biotinylated pentamers (ProImmune) followed by staining with streptavidin BV421 or PE. The multimers used included MHC Class I alleles HLA-A*02, *03, *24, or B*07 plus peptides derived from Gag, Pol, Env, or Nef. PBMCs were incubated with multimer diluted in PBS for 15 minutes at room temperature (pentamer) or 20 minutes at 37°C (tetramer), followed by 20 minutes at room temperature with surface antibodies (with streptavidin for stains with pentamer), along with fixable viability dye to allow for discrimination of dead cells (Thermo Fisher Scientific), and then fixation and permeabilization using the eBioscience Foxp3/Transcription Factor Staining Buffer Set according to manufacturer’s instructions (Thermo Fisher Scientific). Antibodies used are listed in Supplemental Table 3. Cytometer settings were standardized between experiments using Application settings. PBMCs from a single HIV-uninfected individual (processed and cryopreserved on the same day) were run in parallel in most experiments. Flow cytometry panels were optimized using fluorescence-minus-1 controls, and gating for most markers of interest was standardized between experiments by fixing a positive gate according to the proportion of naive (CD45RA^+^CCR7^+^CD27^+^) and/or effector memory CD8^+^ T cells (CD45RA^–^CCR7^–^CD27^–^) from the control donor that expressed the marker. When longitudinal samples were available from a study participant, all samples were stained and run on the flow cytometer on the same experiment day. All experiments characterizing PBMCs from participants with HIV included samples from individuals from all 3 clinical groups. Human flow cytometry data were collected across 5 (Figures 1–3 and Figure 4, D and E), 4 (Figure 4, A and B), 3 (Figure 5, A and D–G, and Figure 6, B–E), or 1 (Figure 4C and Figure 5B) experiments. Data were analyzed using FlowJo v10.1 software (Tree Star Inc.).

### Proliferation by CTV.

Thawed PBMCs in R10 media were rested overnight at 37°C and 5% CO_2_. Cells were then labeled with CTV (Thermo Fisher Scientific) according to the manufacturer’s instructions. One million CTV-labeled cells were stimulated for 5 or 6 days (as indicated) at 37°C with 5% CO_2_ in R10 media containing 0.2 μg/mL HIV peptide pools composed of peptides derived from the Gag, Nef, Env, or Pol HIV proteins (depending on the multimer specificity; supplied by the NIH AIDS Reagent Program), individual HIV- or CMV-specific peptides (if indicated; ProImmune), or αCD3/CD28 per manufacturer’s instructions (ImmuCult human CD3/CD28 T cell activator; StemCell Technologies). After stimulation, the cells were then stained for multimer, surface markers, and intracellular molecules, and they were then analyzed immediately on a flow cytometer.

### Digital droplet PCR quantification of cell-associated HIV DNA.

Genomic DNA was extracted from 1 × 10^7^ PBMCs using the AllPrep DNA/RNA/miRNA Universal Kit (Qiagen). Following nucleic acid isolation, DNA was sheared into 3 kb fragments using the Covaris M220 Focused-Ultrasonicator for 10 minutes. Absolute quantification of the HIV long terminal repeat (LTR) was performed, along with Ribonuclease P (RNaseP) as a cell counter, in a duplex-digital droplet PCR reaction using the RainDrop System (RainDance Technologies). The RainDrop Source instrument was used to generate uniform aqueous droplets (5 picoliter) for each sample on an 8-well microfluidic chip (RainDrop Source Chip). Reactions were carried out in 50 mL volumes with 2 mL 25× Droplet Stabilizer (RainDance Technologies), 25 μL 2× Taqman Genotyping Master Mix (Thermo Fisher Scientific), 900 nm primers, 250 nm probes, and 500 ng nucleic acid. Droplets were thermocycled at 95°C for 10 minutes, 45 cycles of 95°C for 15 seconds, and 59°C for 1 minute, followed by 98°C for 10 minutes. Droplets were then placed in the deck of the RainDrop Sense instrument containing a second microfluidic chip used for single droplet fluorescence detection. Data were analyzed using the RainDrop Analyst Software on a 2-dimensional histogram with FAM intensity on the *x* axis and Vic intensity on the *y* axis. Cell counts were normalized to RNaseP molecules detected, with a no-template control loaded on each chip. HIV-LTR specific primers F522-43 (5′-GCC TCA ATA AAG CTT GCC TTG A-3′; nucleotide positions 522–543 in the HIV-1 HXB2 reference sequence) and R626-43 (5′-GGG CGC CAC TGC TAG AGA-3′; HXB2 626–643) coupled with a FAM-BQ probe (5′-CCA GAG TCA CAC AAC AGA CGG GCA CA 3). RNase P-specific primers (5′-AGA TTT GGA CCT GCG AGC G-3′ and 5′-GAG CGG CTG TCT CCA CAA GT-3′) were coupled with a VIC-MGB probe (5′-TTC TGA CCT GAA GGC TCT GCG CG-3′).

### HIV viral sequencing.

HIV viral RNA was extracted from 140 μL of plasma using the QIAmp Viral RNA Mini Kit (Qiagen). Isolated RNA was treated with TURNO DNase (Thermo Fisher Scientific) and quantified with a NanoDrop Spectrophotometer ND-1000 (Thermo Fisher Scientific). A total of 8 μL viral RNA was reverse transcribed into cDNA with random primers and the SuperScript III First-Strand Synthesis System (Thermo Fisher Scientific) according to the manufacturer’s instructions. Amplification of the HIV gag gene was performed using flanking primers; (5′-AAA TCT CTA GCA GTG GCG CC-3′; HXB2 623–642) and (5′-TGT TGG CTC TGG TCT GCT CT-3′; HXB2 2157–2138). PCR was performed with a Phusion High-Fidelity PCR Kit (New England Biolabs) in 20 μL reactions mixed with 2 μL cDNA and 10 μM primers. Thermal cycling was carried out at 98°C for 30 seconds, 35 cycles of 98°C for 5 seconds, 64°C for 15 seconds, 72°C for 1 minute, and finally 72°C for 10 minutes. Amplicons were purified using the QIAquick PCR purification kit (Qiagen) and Sanger sequenced by MCLAB. Sequences were analyzed with CodonCode Aligner using a base call threshold set to identify HIV escape variants of targeted epitopes. The SLYNTVATL Sequencing Primer is as follows: 5′-ACT AGC GGA GGC TAG AA-3′.

### CRISPR-Cas9 KO of TCF7 in primary human T cells.

Thawed PBMCs in R10 media were rested overnight (incubation conditions for resting or stimulating cells were 37°C and 5% CO_2_) and then prepared for nucleofection based on protocols described previously (86). Briefly, Cas9 RNPs were prepared by first incubating 160 μM tracrRNA with 160 μM *TCF7*-targeting or nontargeting (scramble) guide RNA (Dharmacon) for 30 minutes at 37°C and then incubating this 80 μM RNA product with an equal volume of 40 μM Cas9 (QB3 MacroLab; see Supplemental Table 5 for guide RNA sequences). Up to 20 million PBMCs were pelleted and resuspended in a final volume of 92 μL electroporation buffer P2 (Lonza), combined with 8 μL of the Cas9 RNPs, nucleofected using X-unit large-format cuvettes in a Lonza 4D nucleofector (pulse code EO-100), resuspended in 1 mL R10 buffer, rested for at least 15 minutes, and then brought to 1 million to 2 million PBMCs/mL in R10 buffer plus 500 pg/mL recombinant human IL-7 (rhIL-7) (R&D Systems) and rested again for 4 days. Cells were then harvested, an aliquot was stained for flow cytometry, and the remaining cells were labeled with CTV as described above. To provide fresh feeder cells to the cell culture, autologous (non-CTV-labeled) PBMCs thawed the day prior were depleted of CD8^+^ T cells (using the flow-through from the StemCell EasySep Human CD8 Positive Selection kit), rested at 37°C and 5% CO_2_, and then combined in a 1:1 ratio with the edited CTV-labeled PBMCs with or without αCD3/CD28 stimulatory cocktail and incubated for 5 days (StemCell Technologies). Cells were then harvested and stained for flow cytometry. The proportion of proliferating cells (%CTV^lo^) was calculated as a proportion of total edited cells (which were gated based on positive staining for CD8α and CTV; unedited feeder cells were negative for these markers).

### CRISPR-Cas9 generation of HIV-specific TCR T cells with TCF7 knock-in.

HIV-specific TCR T cells with *TCF7* knock-in were generated using a previously described protocol (59), summarized briefly and with the following modifications. Fresh primary human T cells were isolated and cultured with anti–human CD3/CD28 beads (Dynabeads, Thermo Fisher Scientific), 500 U/mL IL-2 (Proleukin, Prometheus Laboratories), and 5 ng/mL each of IL-7 (R&D Systems) and IL-15 (R&D Systems) for 48–56 hours; then, beads were magnetically separated from the T cells. In parallel, double-stranded homology-directed repair (HDR) DNA templates that contained the following sequences were produced: (a) genes encoding TCRα and TCRβ proteins derived from sequencing of an endogenous HIV-specific CD8^+^ T cell known to recognize the Gag/SL9 peptide presented in the context of HLA-A*02 (using a protocol previously described; ref. 87), plus (b) a sequence for either the full-length human *TCF7* versus truncated *NGFR* gene (see Supplemental Table 5 for all HDR DNA template sequences). The HDR template was combined with RNPs as reported previously (88) and as follows: 500 ng HDR + 0.625 μL crRNA ([stock] = 160μM) + 0.625μL tracrRNA ([stock] = 160μM) + 1 μL poly-L-glutamic acid ([stock] = 125 mg/mL) + 1.25 μL Cas9 ([stock] = 40 μM). A total of 750,000 debeaded T cells was resuspended in 20 μL Lonza electroporation buffer P3, combined with the RNPs + HDR template, and electroporated using a Lonza 4D nucleofector (pulse code EH-115). Cells were rescued in media (Lonza X-Vivo 15 media with 5% FCS, N-acetyl cysteine, and 2-mercaptoethanol) without cytokines for 10 minutes at 37°C and then cultured in media (X-Vivo 15 media with 5% FCS, NAC, and 2-mercaptoethanol) plus 500 U/mL IL-2 for 7 days with exchange of media and IL-2 every 2 days. After 7 days, an aliquot of the cells was harvested and stained for flow cytometry. Edited T cells were CTV labeled and cultured at an effector/target ratio of 1:1 (effector numbers based on the percent of live cells that were HLA-A*02:SL9 pentamer^+^) with an HLA-A*02 cell line as target/antigen presenting cells (T2 cells; ATCC) coated with 1 μM SL9 peptide (ProImmune). Cells were coincubated for 6 days and then stained for evaluation by flow cytometry.

### Statistics.

Nonparametric statistical analysis was performed using GraphPad Prism v7 (GraphPad Software) and STATA v14 (Stata Corp.). Comparisons of parameter measurements between 2 separate groups of participants were assessed with 2-sided Wilcoxon rank sum tests and between 3 or more separate groups were assessed with 2-sided Kruskal-Wallis test followed by Dunn’s multiple comparisons test. In some cases, the same participant contributed data at multiple different time points in the presence and absence of ART (5 individuals had samples available both before and after ART initiation, and 2 individuals had longitudinal samples available after ART). In these cases, linear mixed models with random intercepts were performed to control for clustering by participant, log-transforming outcome variables to satisfy model assumptions. Instances where this approach was used are highlighted in the figure legends. Differences between 2 subsets within individuals were assessed with paired 2-sided Wilcoxon’s signed-rank tests. Correlations between continuous variables were assessed with Spearman’s rank correlations. For all tests, a *P* value less than 0.05 was considered significant.

### Study approval.

For all studies involving human samples, the UCSF Committee on Human Research approved the studies, and participants gave informed, written consent before enrollment. RM were housed and cared for in accordance with standards established by the American Association for Assessment and Accreditation of Laboratory Animal Care (AAALAC) in AAALAC-accredited facilities, and all animal procedures were performed according to protocols approved by the IACUCs of the NIH under ASP LVD26. All experimental procedures were approved by the National Institute of Allergy and Infectious Diseases Division of Intramural Research Animal Care and Use Program as part of the NIH Intramural Research Program (protocols LMM6 and LVD26).

## Author contributions

RLR designed the study and performed all flow cytometry and cell sorting experiments, with assistance from CDTD, WC, CS, and LW. RPS provided MHC Class I monomers. CRISPR-KO experiments were performed by JH and RLR, and CRISPR–knock-in experiments were performed by RLR, FB, and LW, with input from AM and TLR. HIV-specific TCR sequences were provided by RMN, DCD, and RAK. Viral sequencing and reservoir measurements were performed by KAR, and analysis was overseen by SKP. SCOPE and Options samples were provided by SGD, JNM, FMH, and CDP with cohort data management support from RH and MK. Nonhuman primate studies were performed by CES and JCM, with supervision by JMB. The manuscript was written by RLR. PWH, JMM, SGD, and RPS provided scientific guidance throughout.

## Supplementary Material

Supplemental data

## Version 1 12/22/2020

In-Press Preview

## Version 2 02/08/2021

Electronic publication
