# Supplementary material for: TCF-1 regulates HIV-specific CD8+ T cell expansion capacity
Source: JCI Insight. 2021 Feb 8;6(3):e136648. doi: 10.1172/jci.insight.136648 (PMC7934879; doi:10.1172/jci.insight.136648)
Supplement: Supplemental data [file jciinsight-6-136648-s157.pdf]

Supplementary Material:

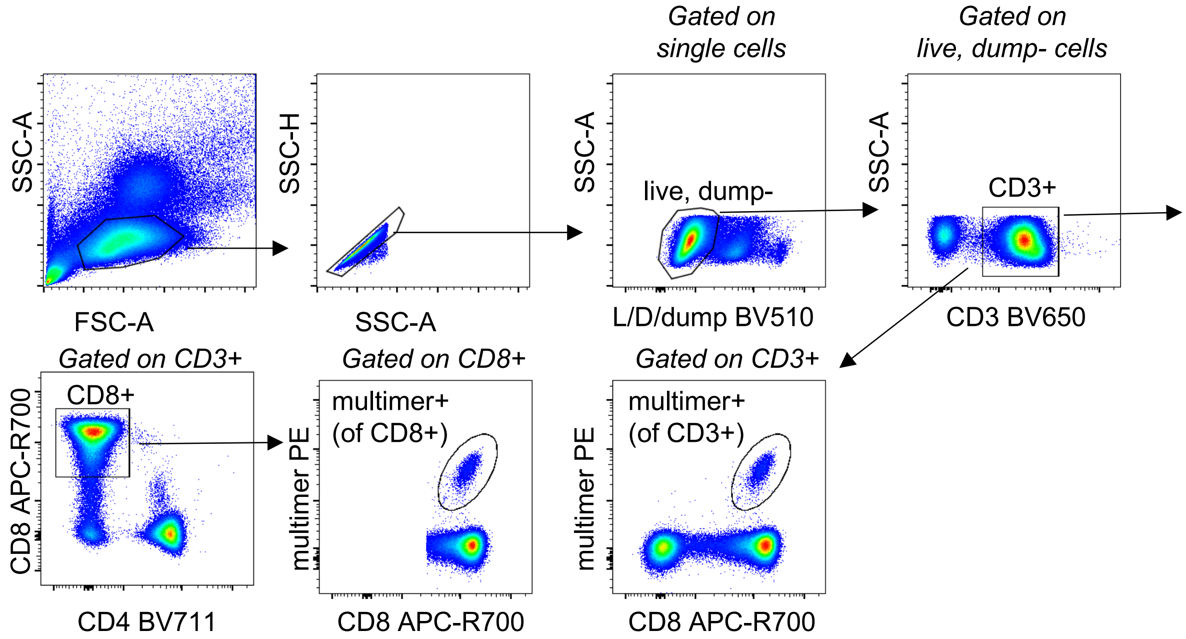

Figure S1. MHC Class I multimer+ HIV-specific CD8+ T cell gating strategy. Dump gate includes staining for CD14, CD19 and TCR-γδ.

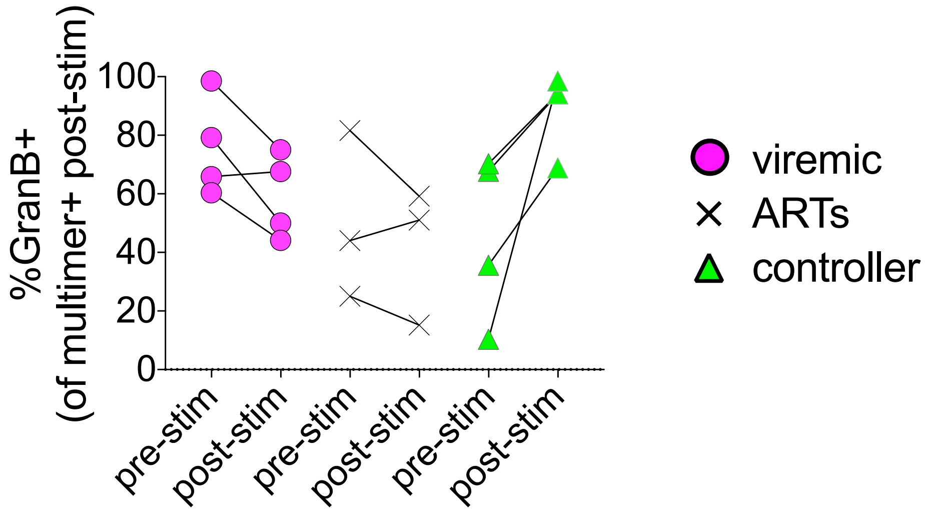

Figure S2. Change in Granzyme B expression after six-day in vitro peptide stimulation of multimer+ HIV-specific CD8+ T cells. Percentage of Granzyme B+ multimer+ cells prior to and after stimulation.

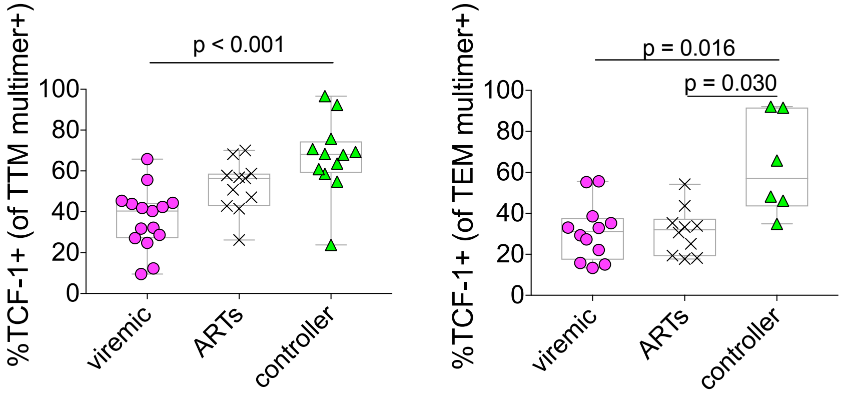

Figure S3. TCF-1 expression amongst TTM and TEM multimer+ HIV-specific CD8+ T cells.
Statistical testing: Linear mixed effects models to account for clustering within participants.

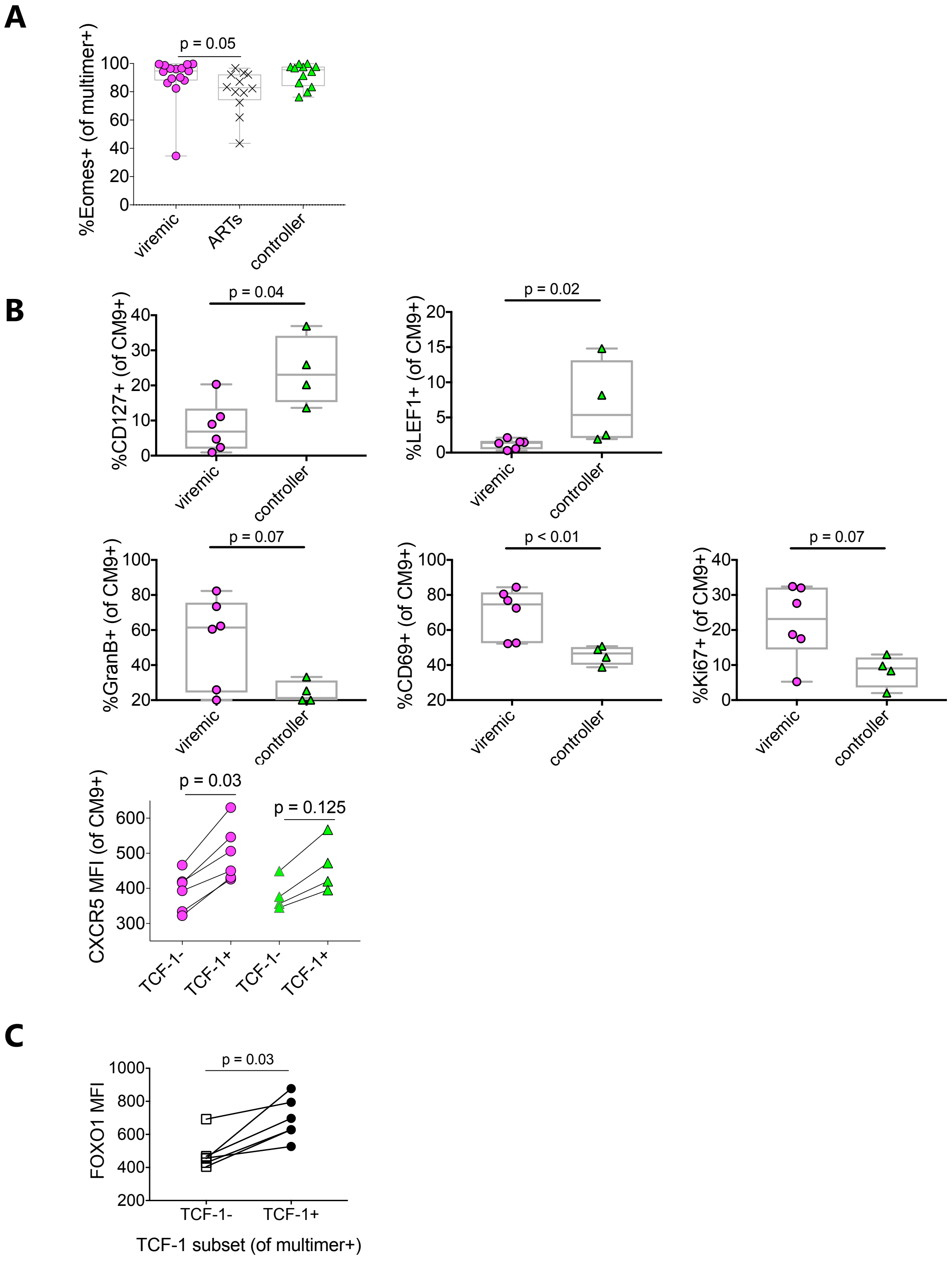

Figure S4. Phenotypes of multimer+ HIV-specific and SIV-specific CD8+ T cells. (**A**) Expression of Eomesodermin (Eomes) in HIV-specific CD8+ T cells. (**B**) Phenotype of SIV-specific CD8+ T cells from viremic and controller animals. (**C**) Expression of FOXO1 in TCF-1+ and TCF-1- subsets of multimer+ HIV-specific CD8+ T cells. Statistical testing: Linear mixed effects models to account for clustering within participants (A), Wilcoxon Rank Sum (B), Wilcoxon Signed Rank (paired data in B; C).

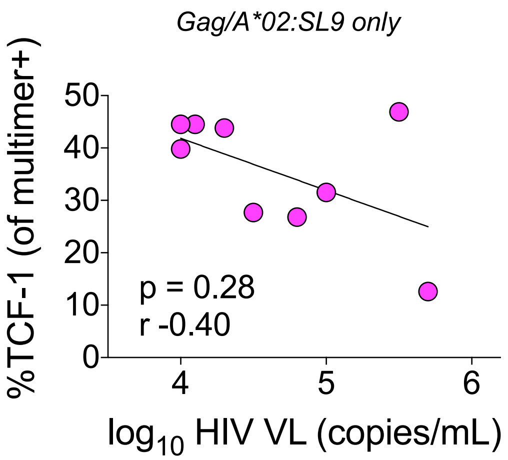

Figure S5. Correlation between TCF-1 expression in multimer+ HIV-specific CD8+ T cells and viral load amongst individuals with documented viral CD8+ T cell escape variants. Statistical testing: Spearman Correlation.

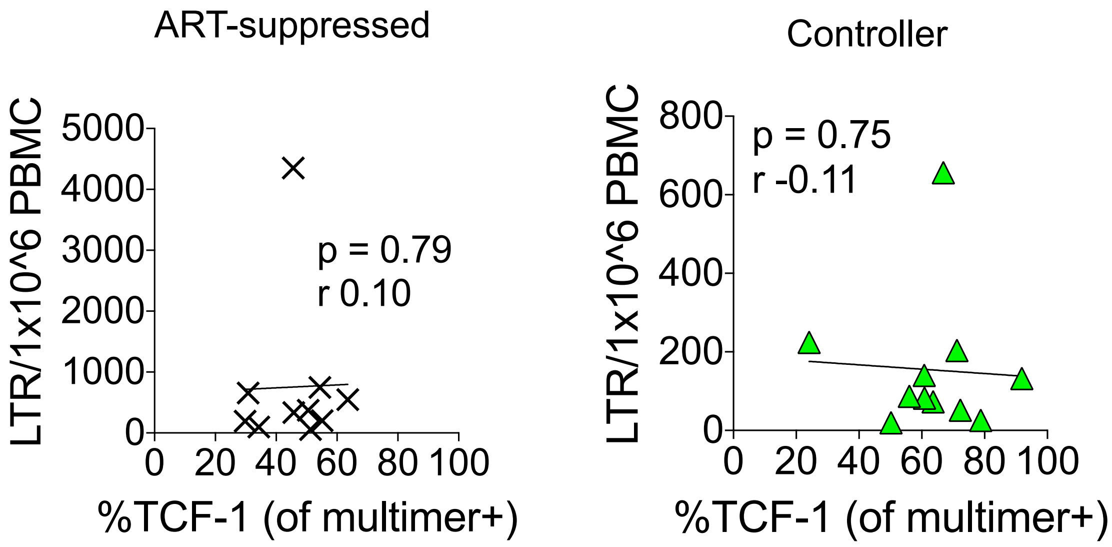

Figure S6. Relationship between TCF-1 expression in HIV-specific CD8+ T cells and HIV DNA levels (long-term repeat [LTR] copies per million PBMC) in ART-suppressed (left) and controller (right) individuals. Statistical testing: Spearman correlation.

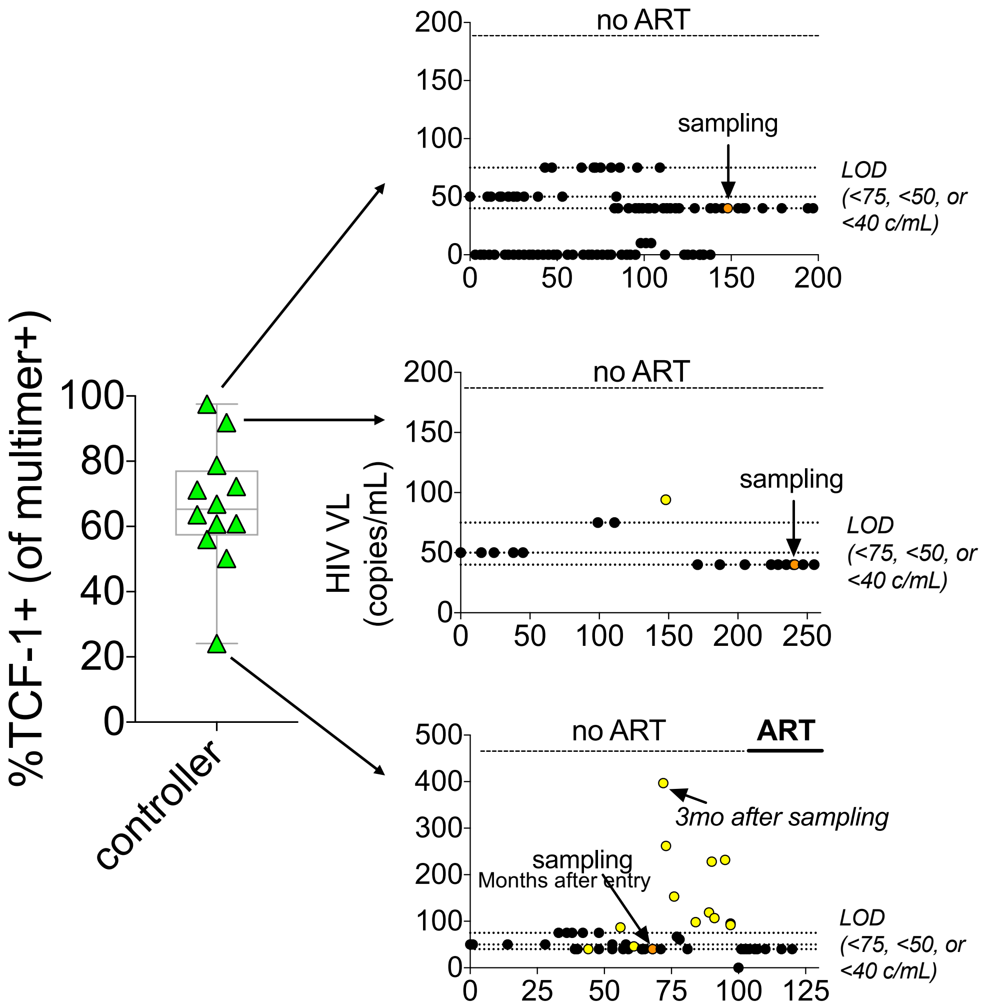

Figure S7. Timing of PBMC in HIV elite controllers with the highest (right panels, top two participants) and lowest (bottom participant) levels of TCF-1 in HIV-specific CD8+ T cells. Detectable HIV viral load measurements noted in yellow. LOD=limit of detection. c/mL=copies/mL.

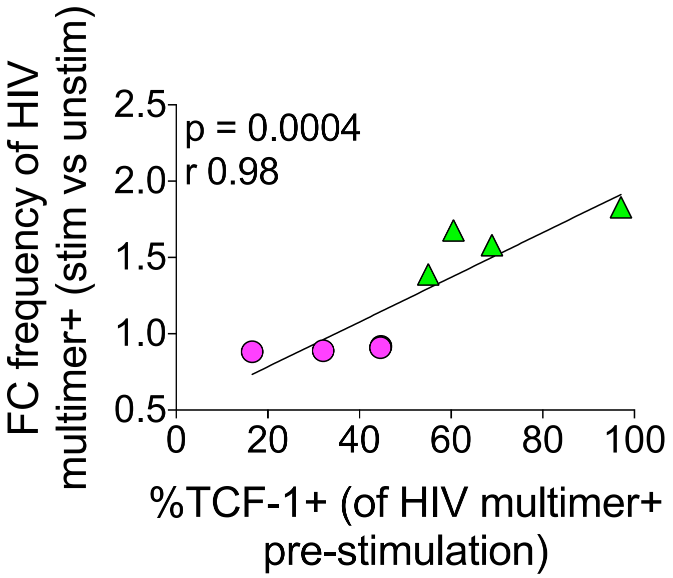

Figure S8. Correlation between TCF-1 expression in HIV-specific CD8+ T cells and their expansion after six-day in vitro peptide stimulation. Fold change [FC] in the frequency of HIV-specific CD8+ T cells (gated on total CD8+ T cells), stimulated versus unstimulated cells.

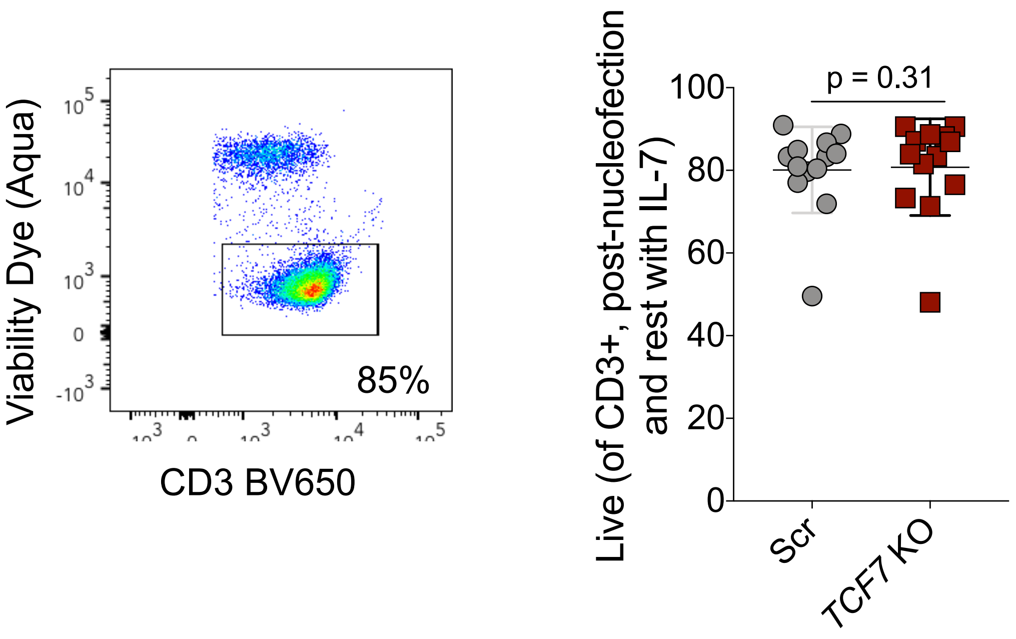

Figure S9. T cell viability after electroporation. Representative flow plot (left) and summary data (left) demonstrating the percent of CD3+ T cells that are viable (i.e., negative for amine-reactive dye staining) 4 days after electroporation with Cas9 plus *TCF7* or scramble (Scr) gRNA (plus IL-7). Statistical testing: Wilcoxon matched-pairs signed rank test.

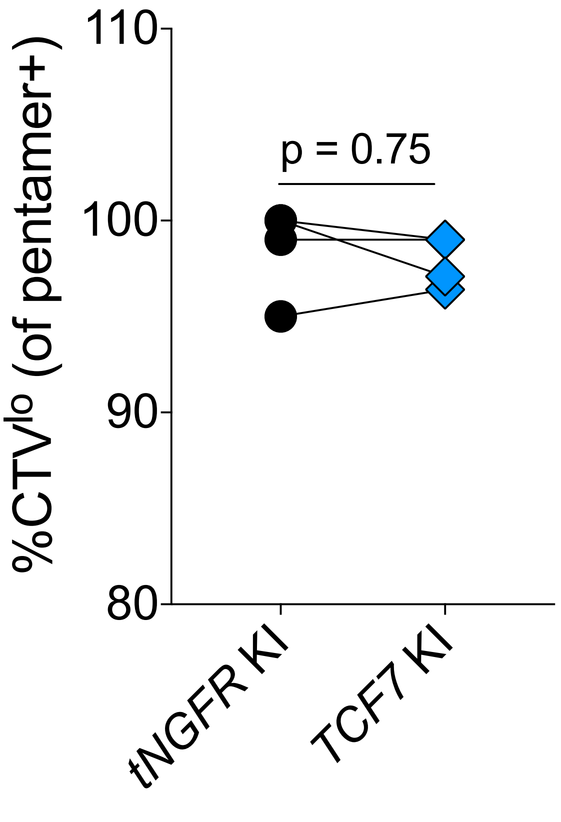

Figure S10. Proliferation of HIV-specific TCR-T cells. Proportion of divided cells with *tNGFR* versus *TCF7* overexpression after 6-day stimulation with cognate peptide-loaded antigen presenting cells. Statistical testing: Wilcoxon Signed Rank test.

	Participant ID (PID)	Gender	Ethnicity	Age (years)	HIV VL (copies /mL)	CD4 count (cells/mm3)	CD4 nadir (cells/mm3)	ART regimen (years on ART)	Protein Target	HLA	Peptide Sequence	Protective HLA (B*27, B*57)
Viremic	1076	Female	White	41	28,122	480	276		Gag	A02	FLGKIWPSYK	
	1079	Male	Latino	52	34,031	381	381		Gag	A02	SLYNTVATL	
	1094	Male	White	40	13,600	376	223		Gag	A02	SLYNTVATL	
	1200	Male	White	52	288,315	265	261		Gag	B07, A02	GPGHKARVL, SLYNTVATL	
	1259	Male	African American	42	92,400	249	249		Gag	A02	SLYNTVATL	
	1284	Male	White	41	9,211	414	414		Gag	A02	SLYNTVATL	
	1348	Male	White	43	29,762	324	324		Gag	B07	GPGHKARVL	
	1435	Male	White	32	66,018	610	402		Gag	A02	SLYNTVATL	
	1503	Male	White	38	10,787	771	426		Gag	A02	SLYNTVATL	Y
	2254	Male	White	39	66,552	471	471		Gag	A24	RYLKDQQLL	
	2303	Male	White	34	500,000	551	478		Gag	A02	SLYNTVATL	
	2412	Male	White	55	21,233	528	544		Gag	B07, A02	GPGHKARVL, SLYNTVATL	
	3611	Male	White	35	986,418	301	291		Gag	A02	FLGKIWPSYK	
ARTs	1098	Male	White	53	<20	563	159	TDF, FTC, ATV/r (6)	Nef	B07	TPGPGVRYPL	
	1200	Male	White	54	<40	862	261	TDF, FTC, ATV/r, RTG (6)	Gag	A02	SLYNTVATL	
	1259	Male	African American	45	<40	338	206	FTC/TDF, ATV/r (2)	Gag	A02	SLYNTVATL	
	1284	Male	White	43	<40	407	271	FTC/TDF, RGV, MVR (2)	Gag	A02	SLYNTVATL	
	2254	Male	White	42, 44	<75, <40	445, 855	435	TDF, FTC, EFV (2, 5)	Env	A24	RYLKDQQLL	Y
	2298	Male	White	63	<40	276	105	TDF, FTC, ATV/r (5)	Gag	A02	FLGKIWPSYK	Y
	2347	Male	White	62	<40	399	75	TDF, FTC, DTG (15)	Nef	B07	TPGPGVRYPL	Y
	2412	Male	White	55, 57	<40, <40	659, 804	544	TDF, FTC, EFV (2, 5)	Gag	B07	GPGHKARVL	
	3098	Female	White	44	<40	512	5	TDF, FTC, ATV/r (2)	Gag	A02	SLYNTVATL	Y
	3510	Male	White	54	<75	400	105	DDI, TDF, FTC, LPV/r, ENF (3)	Pol	B07	SPAIFQSSM	
Controller	1116	Male	White	52	69	584	240		Gag	A02	SLYNTVATL	
	1122	Male	White	37	<40	651	680		Gag	A02	SLYNTVATL	
	1139	Male	Native American	49	46	645	625		Gag	A02	SLYNTVATL	Y
	1349	Male	White	57	<40	742	352		Gag	A02	SLYNTVATL	
	1488	Male	White	33	<40	657	673		Gag	A03	RLRPGGRKR	
	1526	Male	White	40	<40	693	342		Nef	A24	RYPLTFGW	
	1529	Male	White	48	95	876	600		Gag	A02	SLYNTVATL	
	1532	Female	Mixed	46	<75	558	300		Gag	A02	SLYNTVATL	
	1554	Male	White	57	<40	733	400		Gag	A02	FLGKIWPSYK	
	1760	Female	African American	47	<40	596	747		Gag	A02	SLYNTVATL	
	1769	Male	White	48	<40	1098	852		Pol	B07	SPAIFQSSM	
	1783	Male	White	67	<40	648	600		Gag	A02	SLYNTVATL	
	3664	M to F Transgender	Mixed	54	<40	2318	1317		Nef	B07	TPGPGVRYPL	

Table S1. SCOPE participant clinical information. PID, participant ID. TDF, tenofovir disoproxil. FTC, emtricitabine. ATV/r, atazanavir/ritonavir. RGV, raltegravir. MVR, maraviroc. EFV, efavirenz. DTG, dolutegravir. DDI, diadenosine. ENF, enfuviritide.

	Animal ID	SIV Virus	CD4 count (cells/mm3)	SIV VL (copies/mL)
Viremic	CL4C	mac239	47	470000
	CL86	mac239	91	900000
	591	smE543	186	251000
	764	smE543	134	222000
	766	smE543	927	50000
	828	smE543	264	330000
Controller	F98	mac239	560	15
	863	smE660	1245	370
	867	smE660	634	15
	871	smE660	513	15

Table S2. SIV-infected rhesus macaque clinical information.

Anti-human antibodies

Antigen	Clone	Supplier	Fluorochrome
CD14 (dump)	M5E2	Biolegend	BV510
CD19 (dump)	H1B19	Biolegend	BV510
TCR-γδ (dump)	B1	Biolegend	BV510
CD3	SK7	Thermo Fisher Scientific	BV650, PE-Cy5.5
CD4	OKT4	Biolegend	BV650, BV711
CD8α	RPA-T8	BD Biosciences	APC-R700
CD45RA	HI100	Biolegend	APC-Cy7
CCR7	G043H7	Biolegend	BV785
CD27	O323	Biolegend	BV570
CD127	A019D5	Biolegend	BV605
PD-1	EH12.2H7	Biolegend	PE
TIGIT	MBSA43	Thermo Fisher Scientific (Affymetrix)	APC
CD160	BY55	Biolegend	PE-Cy7
2B4	C1.7	Biolegend	FITC
Granzyme B	GB11	Biolegend	FITC
Perforin	B-D48	Biolegend	PE-Cy7
TCF-1	7F11A10	Biolegend	Alexa Fluor 647

Anti-rhesus macaque antibodies

Antigen	Clone	Supplier	Fluorochrome
CD3	SP34-2	BD Biosciences	AF700
CD8	APC-H7	BD Biosciences	SK1
CXCR5	MU5UBEE	Thermo Fisher Scientific (eBioscience)	eFluor 450
CCR7	3D12	BD Biosciences	PE-Cy7
CD95	DX2	Biolegend	PE-Cy5
CD69	FN50	BD Biosciences	PE-Cy7
CD127	HIL-7R-M21	BD Biosciences	PE-CF594
TCF-1	333-966	BD Biosciences	PE
Granzyme B	Gb11	Thermo Fisher Scientific	PE-texas red
Ki-67	B56	BD Biosciences	FITC
LEF1	C12A5	Cell Signaling	unconjugated
(LEF1 secondary)	goat anti-rabbit	BD Biosciences	BV421

Table S3. Antibodies and multimers used for flow cytometry staining.

Participant ID	Major peptide sequence	Secondary detected peptide sequence
1079	SLYNTIAVL	SLYNTIATL
1200	SLYNTVAVL	SLYNTIAVL
1259	SLYNTIAVL	SLYNTVAVL / SLYNTVATL (WT)
1284	SLYNTVAVL	
1435	SLYNTIAVL	SLYNTVAVL
1503	SLYNTIAVL	
1904	SLYNTIAVL	

Table S4. HIV peptide sequence variants of wildtype HLA-A*02:Gag-SL9 9mer (SLYNTVATL) detected in the plasma of HIV viremic participants.

CRISPR *TCF7* KO

Sequence ID	Sequence
Scr gRNA	GTAACGCGAACTACGCGGGT
TCF7 gRNA	TGTGCACTCTGCAATGACCT

CRISPR TCR-T + *TCF7* KI

HLA-A*02 TCR + *tNGFR*:

TTTCAGGTTTCCTTGAGTGGCAGGCCAGGCCTGGCCGTGAACGTTCACTGAAATCATGGCCTCTTGGCCAAGATTGATAGCTTGTGCCTGTCCCTGAGTCCCAGTCC
ATCACGAGCAGCTGGTTTCTAAGATGCTATTTCCCGTATAAAGCATGAGACCGTGACTTGCCAGCCCCACAGAGCCCCGCCCTTGTCCATCACTGGCATCTGGACT
CCAGCCTGGGTTGGGGCAAAGAGGGAAATGAGATCATGTCCTAACCCTGATCCTCTTGTCCCACAGATATCCAGAACCCTGACCCTGCCTCCGGATCCGGAGAGG
GCAGGGGATCTCTCCTTACTTGTGGCGACGTGGAGGAGAACCCCGGCCCCATGagcctcgggctcctgtgctgtggggcctttctctcctgtgggcaggtccagtgaatgctggtgtcactcagaccccaaaattcc
gggtcctgaagacaggacagagcatgacactgctgtgtgcccaggatatgaaccatgaatacatgtactggtatcgacaagacccaggcatggggctgaggctgattcattactcagttggtgagggtacaactgccaaaggagaggtccctgatggctacaatgtc
tccagattaaaaaaacagaatttcctgctggggttggagtcggctgctccctcccaaacatctgtgtacttcTGTGCCAGCAGTTACTCAGGGACGGACTACACCGGGGAGCTGTTTTTTGGAGAAGGCTCTA
GGCTGACCGTACTGGAGGACCTGAAAAACGTGTTCCCACCCGAGGTCGCTGTGTTTGAGCCATCAGAAGCAGAGATCTCCCACACCCAAAAGGCCACACTGGTAT
GCCTGGCCACAGGCTTCTACCCCGACCACGTGGAGCTGAGCTGGTGGGTGAATGGGAAGGAGGTGCACAGTGGGGTCAGCACAGACCCGCAGCCCCTCAAGGAG
CAGCCCGCCCTCAATGACTCCAGATACTGCCTGAGCAGCCGCCTGAGGGTCTCGGCCACCTTCTGGCAGAACCCCCGCAACCACTTCCGCTGTCAAGTCCAGTTCT
ACGGGCTCTCGGAGAATGACGAGTGGACCCAGGATAGGGCCAAACCCGTCACCCAGATCGTCAGCGCCGAGGCCTGGGGTAGAGCAGACTGTGGCTTCACCTCC
GAGTCTTACCAGCAAGGGGTCCTGTCTGCCACCATCCTCTATGAGATCTTGCTAGGGAAGGCCACCTTGTATGCCGTGCTGGTCAGTGCCCTCGTGCTGATGGCTA
TGGTCAAGAGAAAGGATTCCAGAGGCCGGGCCAAGCGGTCCGGATCCGGAcagtgcaccaactatgcgctgctgaaactggcgggcgatgtggaaagcaacccgggcccgATGGGGGCAGGTGCC
ACTGGCCGCGCCATGGACGGGCCGCGCCTGCTGCTGTTGCTGCTTCTGGGGGTGTCCCTTGGAGGTGCCAAGGAGGCATGCCCCACAGGCCTGTACACACACAGC
GGTGAGTGCTGCAAAGCCTGCAACCTGGGCGAGGGTGTGGCCCAGCCTTGTGGAGCCAACCAGACCGTGTGTGAGCCCTGCCTGGACAGCGTGACGTTCTCCGAC
GTGGTGAGCGCGACCGAGCCGTGCAAGCCGTGCACCGAGTGCGTGGGGCTCCAGAGCATGTCGGCGCCATGCGTGGAGGCCGACGACGCCGTGTGCCGCTGCGC
CTACGGCTACTACCAGGATGAGACGACTGGGCGCTGCGAGGCGTGCCGCGTGTGCGAGGCGGGCTCGGGCCTCGTGTTCTCCTGCCAGGACAAGCAGAACACCGT
GTGCGAGGAGTGCCCCGACGGCACGTATTCCGACGAGGCCAACCACGTGGACCCGTGCCTGCCCTGCACCGTGTGCGAGGACACCGAGCGCCAGCTCCGCGAGTG
CACACGCTGGGCCGACGCCGAGTGCGAGGAGATCCCTGGCCGTTGGATTACACGGTCCACACCCCCAGAGGGCTCGGACAGCACAGCCCCCAGCACCCAGGAGC
CTGAGGCACCTCCAGAACAAGACCTCATAGCCAGCACGGTGGCAGGTGTGGTGACCACAGTGATGGGCAGCTCCCAGCCCGTGGTGACCCGAGGCACCACCGAC
AACCTCATCCCTGTCTATTGCTCCATCCTGGCTGCTGTGGTTGTGGGTCTTGTGGCCTACATAGCCTTCAAGAGGTGGAACAGCCGCGCCAAGCGCTCGGGTTCGG
GTGCCACCAACTTCAGCCTGCTGAAGCAGGCCGGCGACGTGGAGGAGAACCCCGGCCCCATGgcatgccctggcttcctgtgggcacttgtgatctccacctgtcttgaatttagcatggctcagacagtcac
tcagtctcaaccagagatgtctgtgcaggaggcagagaccgtgaccctgagctgcacatatgacaccagtgagagtgattattatttattctggtacaagcagcctcccagcaggcagatgattctcgttattcgccaagaagcttataagcaacagaatgcaacagag
aatcgtttctctgtgaacttccagaaagcagccaaatccttcagtctcaagatctcagactcacagctgggggatgccgcgatgtatttcTGTGCTTATAGGAGCGGCCCCCACAATGACATGCGCTTTGGAGCAGGGAC
CAGACTGACAGTAAAACCAAATATCCAGAACCCTGACCCTGCGGTGTACCAGCTGAGAGACTCTAAATCCAGTGACAAGTCTGTCTGCCTATTCACCGATTTTGAT
TCTCAAACAAATGTGTCACAAAGTAAGGATTCTGATGTGTATATCACAGACAAAACTGTGCTAGACATGAGGTCTATGGACTTCAAGAGCAACAGTGCTGTGGCC
TGGAGCAACAAATCTGACTTTGCATGTGCAAACGCCTTCAACAACAGCATTATTCCAGAAGACACCTTCTTCCCCAGCCCAGGTAAGGGCAGCTTTGGTGCCTTCG
CAGGCTGTTTCCTTGCTTCAGGAATGGCCA

HLA-A*-2 TCR + *TCF7*:

TTTCAGGTTTCCTTGAGTGGCAGGCCAGGCCTGGCCGTGAACGTTCACTGAAATCATGGCCTCTTGGCCAAGATTGATAGCTTGTGCCTGTCCCTGAGTCCCAGTCC
ATCACGAGCAGCTGGTTTCTAAGATGCTATTTCCCGTATAAAGCATGAGACCGTGACTTGCCAGCCCCACAGAGCCCCGCCCTTGTCCATCACTGGCATCTGGACT
CCAGCCTGGGTTGGGGCAAAGAGGGAAATGAGATCATGTCCTAACCCTGATCCTCTTGTCCCACAGATATCCAGAACCCTGACCCTGCCTCCGGATCCGGAGAGG
GCAGGGGATCTCTCCTTACTTGTGGCGACGTGGAGGAGAACCCCGGCCCCATGagcctcgggctcctgtgctgtggggcctttctctcctgtgggcaggtccagtgaatgctggtgtcactcagaccccaaaattcc
gggtcctgaagacaggacagagcatgacactgctgtgtgcccaggatatgaaccatgaatacatgtactggtatcgacaagacccaggcatggggctgaggctgattcattactcagttggtgagggtacaactgccaaaggagaggtccctgatggctacaatgtc
tccagattaaaaaaacagaatttcctgctggggttggagtcggctgctccctcccaaacatctgtgtacttcTGTGCCAGCAGTTACTCAGGGACGGACTACACCGGGGAGCTGTTTTTTGGAGAAGGCTCTA
GGCTGACCGTACTGGAGGACCTGAAAAACGTGTTCCCACCCGAGGTCGCTGTGTTTGAGCCATCAGAAGCAGAGATCTCCCACACCCAAAAGGCCACACTGGTAT
GCCTGGCCACAGGCTTCTACCCCGACCACGTGGAGCTGAGCTGGTGGGTGAATGGGAAGGAGGTGCACAGTGGGGTCAGCACAGACCCGCAGCCCCTCAAGGAG
CAGCCCGCCCTCAATGACTCCAGATACTGCCTGAGCAGCCGCCTGAGGGTCTCGGCCACCTTCTGGCAGAACCCCCGCAACCACTTCCGCTGTCAAGTCCAGTTCT
ACGGGCTCTCGGAGAATGACGAGTGGACCCAGGATAGGGCCAAACCCGTCACCCAGATCGTCAGCGCCGAGGCCTGGGGTAGAGCAGACTGTGGCTTCACCTCC
GAGTCTTACCAGCAAGGGGTCCTGTCTGCCACCATCCTCTATGAGATCTTGCTAGGGAAGGCCACCTTGTATGCCGTGCTGGTCAGTGCCCTCGTGCTGATGGCTA
TGGTCAAGAGAAAGGATTCCAGAGGCCGGGCCAAGCGGTCCGGATCCGGAcagtgcaccaactatgcgctgctgaaactggcgggcgatgtggaaagcaacccgggcccgATGCCGCAGCTGGAT
TCTggcGGAGGAGGAgcgggcGGAGGAGATGATctcggcgcgccgGATGAGCTGCTGGCCTTCCAGGATGAAGGCGAGGAGCAGGATGACAAGAGCCGAGATAGCGCCGC
CGGTCCTGAGCGCGACCTGGCCGAGCTCAAGTCGTCGCTTGTGAACGAATCCGAGGGAGCAGCCGGAGGAGCAGGAATCCCGGGAGTTCCGGGAGCCGGCGCCG
GAGCCCGAGGCGAGGCCGAAGCTCTTGGACGAGAACACGCTGCGCAGAGACTCTTCCCGGACAAACTTCCAGAGCCCCTGGAGGACGGCCTGAAGGCCCCGGAG
TGCACCAGCGGCATGTACAAAGAGACCGTCTACTCCGCCTTCAATCTGCTCATGCATTACCCACCTCCTTCGGGAGCAGGACAGCACCCTCAGCCGCAGCCTCCGC
TGCACAAGGCCAATCAGCCTCCTCACGGTGTCCCTCAACTCTCTCTCTACGAACATTTCAACAGCCCACATCCCACCCCTGCACCTGCGGACATCAGCCAGAAGCA
AGTTCACAGGCCTCTGCAGACCCCTGACCTCTCTGGCTTCTACTCCCTGACCTCAGGCAGCATGGGGCAGCTCCCCCACACTGTGAGCTGGTTCACCCACCCATCCT
TGATGCTAGGTTCTGGTGTACCTGGTCACCCAGCAGCCATCCCTCACCCGGCCATTGTGCCTCCTTCAGGGAAGCAGGAGCTGCAGCCTTTCGACCGCAACCTGAA
GACACAAGCAGAGTCCAAGGCAGAGAAGGAGGCCAAGAAGCCAACCATCAAGAAGCCCCTCAATGCCTTCATGCTGTACATGAAGGAGATGAGAGCCAAGGTCA
TTGCAGAGTGCACACTTAAGGAGAGCGCTGCCATCAACCAGATCCTGGGCCGCAGGTGGCACGCGCTGTCGCGAGAAGAGCAGGCCAAGTACTATGAGCTGGCC
CGCAAGGAGAGGCAGCTGCACATGCAGCTATACCCAGGCTGGTCAGCGCGGGACAACTACGGGAAGAAGAAGAGGCGGTCGAGGGAAAAGCACCAAGAATCCA
CCACAGGAGGAAAAAGAAATGCATTCGGTACTTACCCGGAGAAGGCCGCTGCCCCAGCCCCGTTCCTTCCGATGACAGTGCTCCGCGCCAAGCGCTCGGGTTCGG
GTGCCACCAACTTCAGCCTGCTGAAGCAGGCCGGCGACGTGGAGGAGAACCCCGGCCCCATGgcatgccctggcttcctgtgggcacttgtgatctccacctgtcttgaatttagcatggctcagacagtcac
tcagtctcaaccagagatgtctgtgcaggaggcagagaccgtgaccctgagctgcacatatgacaccagtgagagtgattattatttattctggtacaagcagcctcccagcaggcagatgattctcgttattcgccaagaagcttataagcaacagaatgcaacagag
aatcgtttctctgtgaacttccagaaagcagccaaatccttcagtctcaagatctcagactcacagctgggggatgccgcgatgtatttcTGTGCTTATAGGAGCGGCCCCCACAATGACATGCGCTTTGGAGCAGGGAC
CAGACTGACAGTAAAACCAAATATCCAGAACCCTGACCCTGCGGTGTACCAGCTGAGAGACTCTAAATCCAGTGACAAGTCTGTCTGCCTATTCACCGATTTTGAT
TCTCAAACAAATGTGTCACAAAGTAAGGATTCTGATGTGTATATCACAGACAAAACTGTGCTAGACATGAGGTCTATGGACTTCAAGAGCAACAGTGCTGTGGCC
TGGAGCAACAAATCTGACTTTGCATGTGCAAACGCCTTCAACAACAGCATTATTCCAGAAGACACCTTCTTCCCCAGCCCAGGTAAGGGCAGCTTTGGTGCCTTCG
CAGGCTGTTTCCTTGCTTCAGGAATGGCCA

Table S5. Guide RNA and HDR template sequences for CRISPR-Cas9 knock-out and knock-in experiments.
